# Preparation of Group I Introns for Biochemical Studies and Crystallization Assays by Native Affinity Purification

**DOI:** 10.1371/journal.pone.0006740

**Published:** 2009-08-27

**Authors:** Quentin Vicens, Anne R. Gooding, Luis F. Duarte, Robert T. Batey

**Affiliations:** 1 Department of Chemistry and Biochemistry, University of Colorado, Boulder, Colorado, United States of America; 2 Howard Hughes Medical Institute, University of Colorado, Boulder, Colorado, United States of America; 3 Mount Sinai School of Medicine, New York, New York, United States of America; University Paris 7, France

## Abstract

The study of functional RNAs of various sizes and structures requires efficient methods for their synthesis and purification. Here, 23 group I intron variants ranging in length from 246 to 341 nucleotides—some containing exons—were subjected to a native purification technique previously applied only to shorter RNAs (<160 nucleotides). For the RNAs containing both exons, we adjusted the original purification protocol to allow for purification of radiolabeled molecules. The resulting RNAs were used in folding assays on native gel electrophoresis and in self-splicing assays. The intron-only RNAs were subjected to the regular native purification scheme, assayed for folding and employed in crystallization screens. All RNAs that contained a 3′ overhang of one nucleotide were efficiently cleaved off from the support and were at least 90% pure after the non-denaturing purification. A representative subset of these RNAs was shown to be folded and self-splicing after purification. Additionally, crystals were grown for a 286 nucleotide long variant of the *Clostridium botulinum* intron. These results demonstrate the suitability of the native affinity purification method for the preparation of group I introns. We hope these findings will stimulate a broader application of this strategy to the preparation of other large RNA molecules.

## Introduction

**Figure 1:**
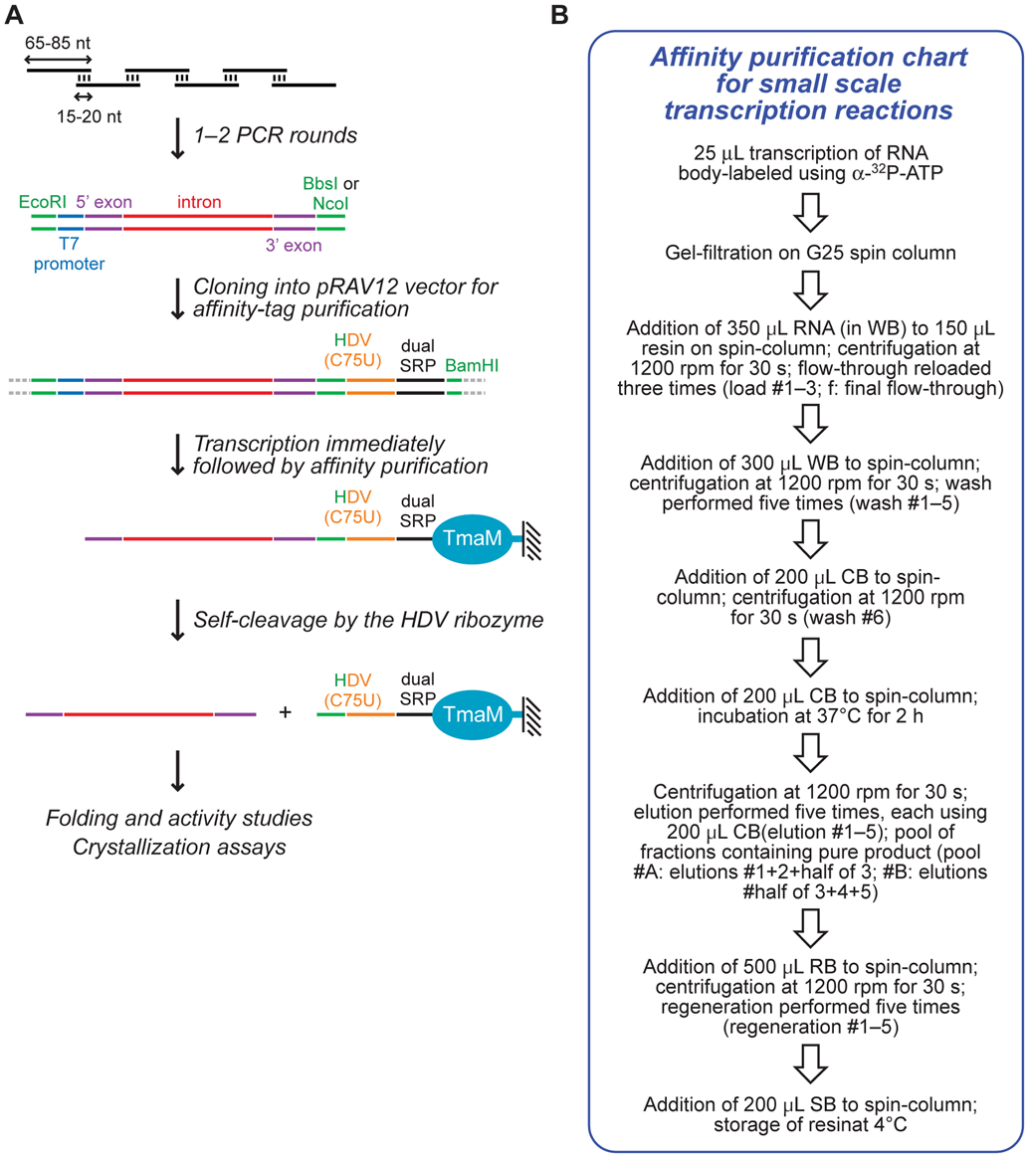
A general strategy to synthesize and purify group I introns within the corresponding exons. (A) General scheme of synthesis by PCR from overlapping primers followed by native purification of the transcribed RNA. The variant shown contains a group I intron (red), 5′ and 3′ exons (purple), a T7 promoter (blue), and restriction sites suitable for cloning (green). The mutated HDV, the dual SRP tag, and the *Tma*M protein coupled to a support are shown in orange, black, and cyan, respectively. Note that the NcoI restriction site was engineered within the 5′ end of the HDV mutant [3], while the BbsI restriction site is no longer present in the final RNA. (B) Affinity purification chart for small-scale transcription reactions developed in this study (WB: wash buffer; CB: cleavage buffer; SB: storage buffer; RB: regeneration buffer; see Materials and Methods section).

The rapid increase in the discovery of RNA molecules as key players in biological mechanisms [1], [2] requires fast and efficient methods to prepare natively folded molecules suitable for biochemical analyses of their structures and functions. Such a method has been developed that employs non-denaturing affinity separation mediated by a tag engineered on the 3′ end of the RNA of interest [3]. This additional sequence comprised a mutant of the genomic hepatitis delta virus (HDV) ribozyme that self-cleaves only in the presence of imidazole [4], followed by two stem-loops derived from the signal recognition particle (SRP) RNA [5]. After transcription completion, the RNA was separated from the reaction through binding of the dual SRP tag to its protein partner (a domain of the *T. maritima* Ffh protein (*Tma*M)) immobilized on a resin, and then recovered by elution in the presence of imidazole (**Figure 1A**). This technique facilitated the crystallization of several small- and medium-size RNAs (<160 nucleotides (nt)), in particular the purine- and the magnesium-sensing riboswitches [6], [7].

From the outset, the application of this purification technique to a broader variety of molecules proved to be limited by recurrent technical difficulties such as the precipitation of the *Tma*M-domain protein at low salt concentrations, the short lifetime of the resin, the degradation of RNA molecules in the presence of imidazole, and the poor cleavage of some sequences by HDV. These shortcomings were addressed in the next generation of this native affinity purification technique [8]. In the meantime, we sought to investigate whether RNA molecules longer than 200 nt could be purified by the first-generation technique in quality suitable for both biochemical and crystallization assays.

Here, we report the PCR-based construction from overlapping primers of six group I intron genes belonging to four different organisms, as well as the native affinity purification of the corresponding RNAs (246–341 nt) using the first-generation method. We present a version of the original protocol that allowed purification of small-scale transcription reaction products incorporating radiolabeled nucleotides. These RNAs were subjected to folding assays and catalytic activity assays. We also applied the standard native affinity purification method to 12 sequence variants of a 287 nt long group I intron from the bacterium *Clostridium botulinum*. Crystals were obtained for one of the circularly permuted variants and were amenable to X-ray diffraction analysis. Finally, we present the limitations to the application of this method that were encountered over the course of this study. Overall, our results indicate that RNAs ranging in size from 246–341 nt, ending with a 3′ overhang of one nucleotide, and purified using the native affinity technique, could be successfully used in a wide range of biochemical and structural assays.

## Results

### Selection, design and PCR-amplification of variants

**Figure 2:**
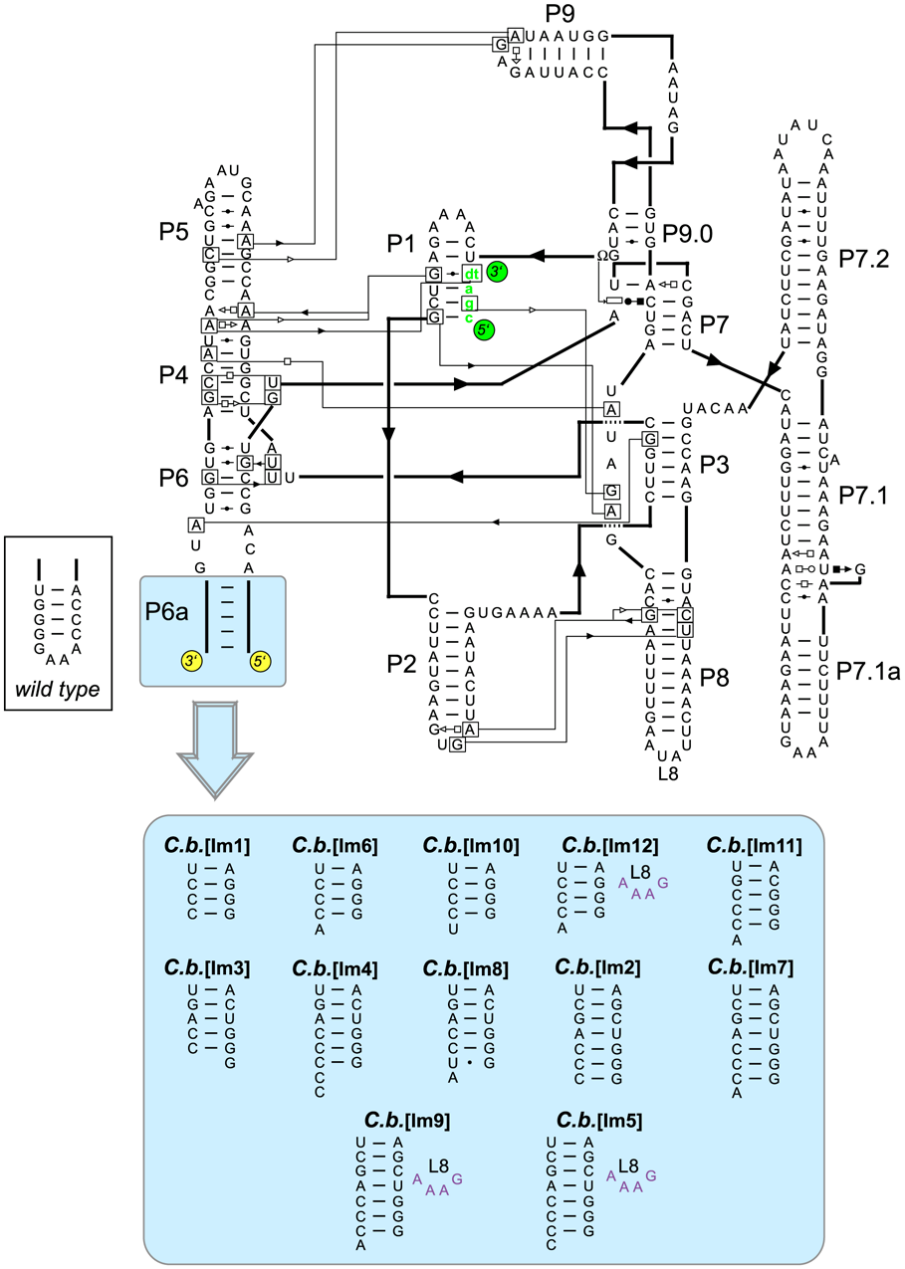
Predicted secondary structure of the *Clostridium botulinum* intron variants used in this study. Base pairing within long-range interactions and structural motifs is shown using the Leontis and Westhof nomenclature [40], where circles denote interactions involving the Watson-Crick face, squares the Hoogsteen face, and triangles the sugar edge. The different sequences of the P6a stem in the twelve mutants are shown in the blue box (wild type sequence and topology shown in the inset for comparison). The substrate oligonucleotide is shown in green.

The six group I intron RNAs employed in this study originated from bacteria (*Anabaena sp. PCC7120, A.s.; Clostridium botulinum, C.b.; Derbesia marina, D.m.; Bryopsis plumosa, B.p.*) [9]–[11], fungus (*Scytalidium dimidiatum, S.d.*) [12], and phage (bacteriophage T4, *td*) [13]. A total of 20 DNA templates of various lengths (280–683 nt) were designed to encode these introns either alone (designated by “[I]” for “intron”; **Table 1**) or embedded in exons (designated by “[P]” for precursor; **Table 1**). For the precursors, exons were either kept at their natural lengths (*A.s., D.m., B.p.*) or shortened to 75 nucleotides (*td, S.d., C.b.*). Variants of the *td* and *C.b.* introns were also designed for crystallization purposes. *td*[Im] contained a mutated P6a domain (Eric Westhof, pers. comm.). The *C.b.*[Im1–12] variants corresponded to 12 variants of the *C.b.* group I intron that possessed 5′ and 3′ ends circularly permuted to the P6a region and further engineered in both the P6a helix and loop L8 (**Figure 2**). Circular permutations of various group I introns had been studied earlier [14], [15].

**Table 1:** Summary of the outcomes of the PCR amplification and of the native purification for each RNA.

Organism [gene type]^1^	Variant abbreviation^2^	Variant description	Total length of variant ligated to pRAV12 (base pairs)^3^	Method of synthesis	DNA polymerase tested^4^	Number of overlapping DNA primers
*Anabaena sp. PCC7120 [tRNA^Leu^ (UAA)]*	*A.s.*[P]	5′ exon: 34 nt; intron: 250 nt; 3′ exon: 45 nt (total: 339 nt)	374	PCR from plasmid	Herculase	n.a.
	*A.s.*[Pm1]	5′ exon: 36 nt; intron: 250 nt; 3′ exon: 45 nt (total: 341 nt)	376	PCR from overlapping primers	Taq*, Pwo, Herculase	8
*Derbesia marina [tRNA^Leu^(UAA)]*	*D.m.*[P]	5′ exon: 35 nt; intron: 167 nt; 3′ exon: 50 nt (total: 252 nt)	287	PCR from overlapping primers	Taq	6
*Bryopsis plumosa [tRNA^Leu^ (UAA)]*	*B.p.*[P]	5′ exon: 35 nt; intron: 206 nt; 3′ exon: 51 nt (total: 292 nt)	327	PCR from overlapping primers	Taq	7
*Bacteriophage T4 [td]*	*td*[Pm1]	5′ exon: 30 nt; intron: 253 nt; 3′ exon: 45 nt (total: 328 nt)	362	PCR from overlapping primers	Taq, Herculase*	7
	*td*[Im1]	intron: 246 nt	280	PCR from overlapping primers	Herculase	5
	*td*[I]	intron: 257 nt	291	PCR from overlapping primers	Herculase	5
*Scytalidium dimidiatum [SSU]*	*S.d.*[P]	5′ exon: 30 nt; intron: 393 nt; 3′ exon: 45 nt (total: 468 nt)	503	PCR from overlapping primers	Taq, Pwo, Herculase	10
*Clostridium botulinum [tmRNA]*	*C.b.*[P]	5′ exon: 338 nt; intron: 287 nt; 3′ exon: 24 nt (total: 649 nt)	683	PCR from plasmid	Herculase	n.a.
	*C.b.*[Im1–12]	intron: 283–290 nt	317–324	PCR from plasmid	Herculase	n.a.

Fraction of clones containing the expected sequence (out of the total number tested)	Yield of cleavage from support (%)^5^	Purity (%)^5^	Amount of purified RNA (nmoles/mL of transcription reaction)	Successfully purified using the nondenaturing method	GenBank accession number	Reference
1/1	83*	n.a.	n.d.	no	AJ228705	[11], [38]
1/3	38	99	n.d.	yes	AJ228705	
1/4	52*	99	n.d.	yes	n.a.	K. Kowallik, pers. comm.
1/4	31*	99	n.d.	yes	M61159	[9]
7/15	36*	91	n.d.	yes (*td*[Pm1b]); no (*td*[Pm1a)]	AF158101	[13], [37]
4/5	n.d.	n.d.	n.d	yes	AF158101	
1/2	n.d.	n.d.	n.d.	yes	AF158101	
0	n.a.	n.a.	n.a.	n.a.	AF258603	[12]
1/1	n.a.	n.a.	n.a.	no	AM412317	[10]
1/1	3 (*C.b.* [Im1])	<5 (*C.b.*[Im1])	n.a.	no		
	18 (*C.b.*[Im2])	85 (*C.b.*[Im2])	0.5	yes (but low yield)		
	5 (*C.b.*[Im3])	75 (*C.b.*[Im3])	0.5	no		
	12 (*C.b.* [Im4])	80 (*C.b.* [Im4])	0.5	no		
	63 (*C.b.* [Im5])	>90 (*C.b.* [Im5])	2	yes		
	30 (*C.b.* [Im6])	>90 (*C.b.* [Im6])	17	yes		
	73 (*C.b.* [Im7])	>90 (*C.b.* [Im7])	22	yes		
	79 (*C.b.* [Im11])	70 (*C.b.* [Im11]; small-scale); >90 (*C.b.* [Im11]; large scale)	10	yes		
	>50.0 (*C.b.* [Im8]; *C.b.* [Im9]; *C.b.* [Im10]; *C.b.* [Im12])	>90 (*C.b.* [Im8]; *C.b.* [Im9]; *C.b.* [Im10]; *C.b.* [Im12])	24 (*C.b.*[Im8]); n.d. (*C.b.*[Im9]); 20 (*C.b.*[Im10]); 8 (*C.b.*[Im12])	yes		

1 *tRNA (anticodon); SSU*: small ribosomal subunit; *td*: thymidylate synthase; *tmRNA*: transfer-messenger RNA.

2 P: precursor; Pm1: precursor containing mutations in the tRNA acceptor stem (*A.s.*) or in P6a (*td*); I: intron; Im1: intron containing mutations in P6a; Im1–12: variants #1 to #12 containing mutations in P6a.

3 Comprises 35 additional base pairs to the variant sequence, corresponding to restriction sites and a T7 polymerase promoter.

4 When two or more polymerases were tested, a star indicates which one was successful.

5 See Materials and Methods for calculation details; n.d.: not determined; n.a.: not applicable.

**Figure 3:**
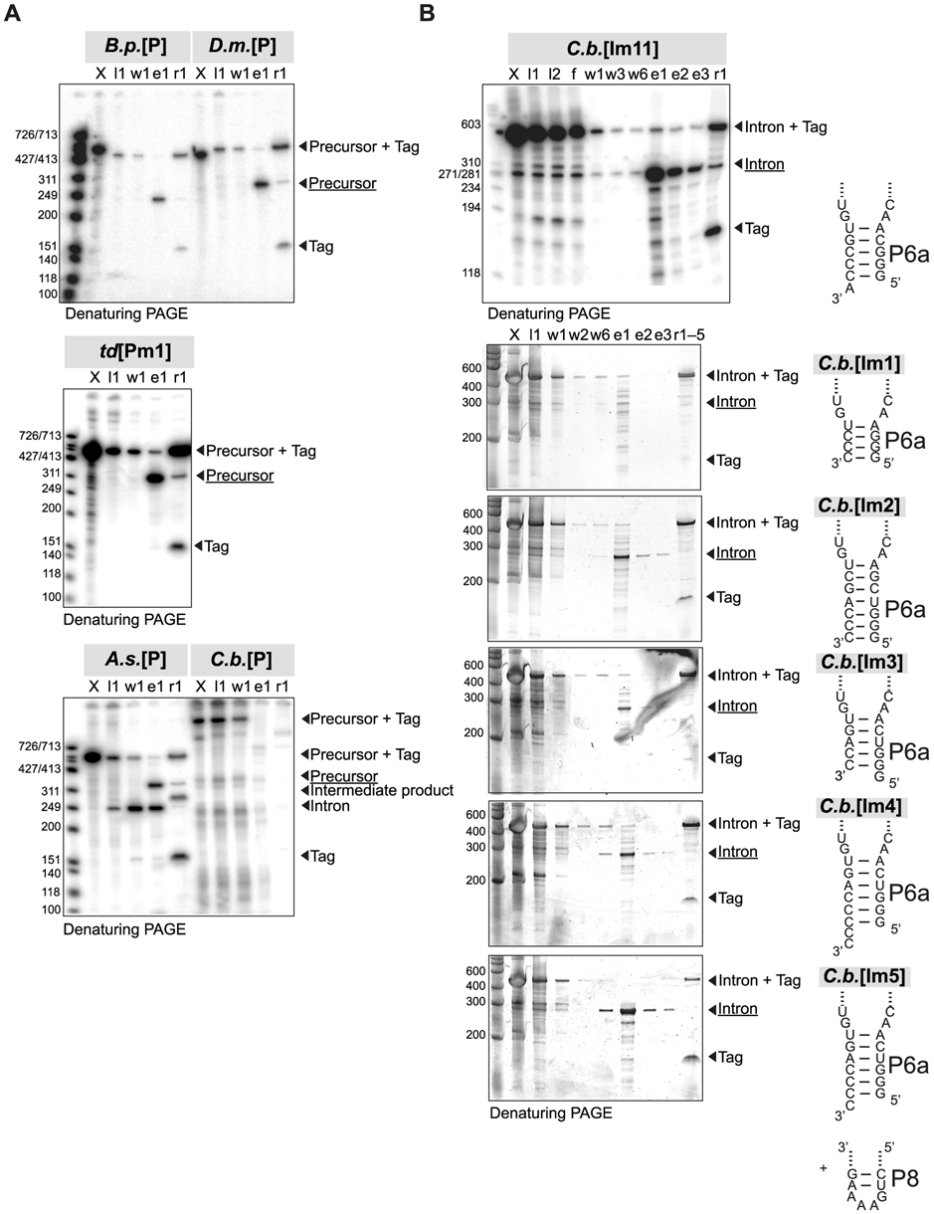
Native affinity purification of various RNAs as visualized by denaturing PAGE. (A) Purification of precursor RNAs each containing an intron with both exons. The intermediate product of the *A.s.*[P] intron could be the 5′ exon followed by the intron (product of hydrolysis at the 3′ splice site), or the intron followed by the 3′ exon (product of self-splicing). Labels above each lane refer to purification steps detailed in Figure 1B (X: transcription reaction; l1–2: load #1–2; f: final flow-through; w1–7: wash #1–7; e1–3: elution #1–3; r1–5: regeneration #1–5). The various products are indicated on the right side of each gel. In this and subsequent figures, the expected product is underlined. (B) Purification of *C.b.* intron variants (nomenclature as in panel A). Note that all the gels in panel A and the gel of *C.b.*[Im11] in panel B show radiolabeled RNA whereas the gels of *C.b.*[Im1–5] in panel B depict unlabeled RNA (i.e. the gels were stained before being scanned). These gels were not adjusted for contrast.

* Estimation based on the amount of cleaved off and uncleaved RNAs present in lanes e1 and r1 only (Fig. 3A; see Materials and Methods).

Whereas amplifications of the *C.b.*[P] and *C.b.*[Im1–12] variants were performed by PCR from a plasmid template, all variants from the *A.s.*, *D.m.*, *B.p.*, and *td* introns were PCR-amplified from 5–8 primers that were 65–85 nt long and overlapping by 15–20 nt (**Tables 1** **and** **S1**). These variants were obtained using either the Taq or Herculase DNA polymerases, after one or two rounds of PCR (**Table 1** **; Figure S1**). Attempts to synthesize the *S.d.*[P] template at different temperatures (50–65°C) and using the three different DNA polymerases were unsuccessful; the only achievement was a PCR product containing the first 200 nt of the expected sequence (**Figure S1**). The failure of the *S.d.*[P] template to be engineered by this technique could be accounted for by a combination of its length (503 base pairs)—thus increasing the number of overlapping primers to 10 (**Table 1**)—and of its high GC content (68%; the GC content of the *A.s.*[P], *D.m.*[P], *B.p.*[P], *td*[P] templates is 43%, 27%, 26% and 42%, respectively).

PCR-amplification from overlapping primers randomly generated mutations within or close to the overlapping regions. Typically, only 25% of the clones sequenced following ligation to the pRAV12 plasmid contained the expected sequence (**Table 1**). Using higher amounts of template (100–1000 ng) [16] and site-directed mutagenesis with primers to reintroduce the correct sequence [17] offered potential solutions that were not pursued here. Instead, switching to a higher fidelity polymerase like Herculase (Stratagene) increased the percentage of correct sequences to 50%, even when performing the two-round PCR amplification procedure (compare for example *td*[Pm1] to *td*[I]; **Table 1**). Finally, two mutants of the *td*[Pm1] template (*td*[Pm1a] and *td*[Pm1b]) that resulted from random PCR errors were further transcribed and purified. The corresponding RNAs were shown to retain some activity (see below). In summary, the templates that were optimally synthesized were about 300 nucleotides long (corresponding to five overlapping primers) and were amplified in one round using a high-fidelity DNA polymerase.

### Native purification of 246–341 nucleotide-long RNAs

The preparation of the *Tma*M protein and its coupling to a support to generate the affinity resin were performed as described [3]. However, the original purification procedure [3] was here optimized for small-scale transcription reactions by employing spin columns and a table top microcentrifuge (**Figure 1B**). These adjustments made it possible to purify six transcription reactions of 25 µL each in about 3 hours, using 150 µL of affinity resin. This implementation was performed independently of the development of a similar one based on the second generation purification method [8].

More than 75% of the full-length transcript is typically retained on the resin (as estimated by comparing the number of counts of the corresponding band in the flow-through lane to that in the transcription reaction lane; respectively lanes f and X/G in Figures 3B and S3). In the end, 15 out of the 21 natural and mutant RNAs were eluted with an average yield of 53% (the standard deviation is 17%; Table 1), to a purity >90% (up to 99% for *A.s.*[Pm1], *B.p.*[P], and *D.m*.[P]; **Table 1**). These values are roughly in line with earlier estimates for 49-nt and 160-nt long RNAs purified on a large scale [3]. Although some moderate (10–30%) and extreme (>80%) cleavage yields may be a consequence of the assumptions made for the calculation (see Materials and Methods), the majority of the cleavage yields can be correlated to the secondary structure at the cleavage site, as suggested earlier [8], regardless of the scale of the purification. In particular, all sequences possessing a 3′ overhang of at least one nucleotide are cleaved with efficiencies >30% (e.g., *B.p.*[P], *D.m.*[P], *A.s.*[P], *C.b.*[Im5]), while variants possessing no overhang (e.g., *C.b.*[Im1], *C.b.*[Im2]) or a 5′ overhang (*C.b.*[Im3]) give moderate to low (<10%) cleavage yields (**Table 1** **,** **Figure 3** **)**. *C.b.*[Im4] was an exception to this rule, as only 12% were cleaved by HDV, in spite of the two-nucleotide 3′ overhang (**Table 1** **;** **Figure 3B**). As a rationale for this observation, we propose that for a large fraction of these molecules, the two dangling cytosines would pair with two of the guanines at positions 38–40 in the joining region J1/4 of the HDV. The formation of such long-range interactions would likely interfere with folding of the ribozyme active site [18], [19]. Hence, the subsequent *C.b.*[Im6–12] variants all incorporated a 3′ dangling adenine or uridine (**Figure 2**) and were cleaved off by HDV with >50% efficiencies (**Table 1** **;** **Figures 3B** **and** **S2**). Typically, the higher the cleavage yield, the higher the purity (**Table 1**), as a high cleavage yield leads to a higher fraction of the expected RNA among degradation products that result from the incubation step in the presence of imidazole [**3**].

The efficiency of the native purification method was compared to that of a standard denaturing gel purification technique for a subset of the RNAs (See Materials and Methods). The number of counts per minute (cpm) measured using a scintillation counter for 1.0 µL of the natively purified radiolabeled *A.s.*[Pm1] and *td*[Pm1b] RNA solutions was in the same 50,000–100,000 range as that obtained for RNAs purified using the conventional denaturing technique (side-by-side comparison for *A.s.*[Pm1] and comparison with other radiolabeled group I intron variants transcribed in similar conditions [23]). Additionally, molar amounts per mL of transcription reaction were estimated for the *C.b.* variants prepared using the large-scale affinity purification. These were typically comprised between 2.0 nmoles/mL (*C.b.*[Im5]; Table 1) and 24 nmoles/mL (*C.b.*[Im8]; Table 1), corresponding to 1.0–12.0 mg of RNA purified from a 5.0 mL transcription reaction. Here as well, these yields were similar to that obtained from the denaturing technique: 3.0–14.0 nmoles/mL of transcription reaction, as estimated for two batches of the *C.b.*[Im1] RNA and one batch of the *C.b.*[Im3] RNA. In short, both the small and the large-scale native purification methods are competitive with a conventional denaturing technique for a quantitative preparation of RNA. All the successfully purified RNAs (**Table 1**) were further used in biochemical assays aimed at characterizing their folding, catalytic, and structural properties.

**Figure 4:**
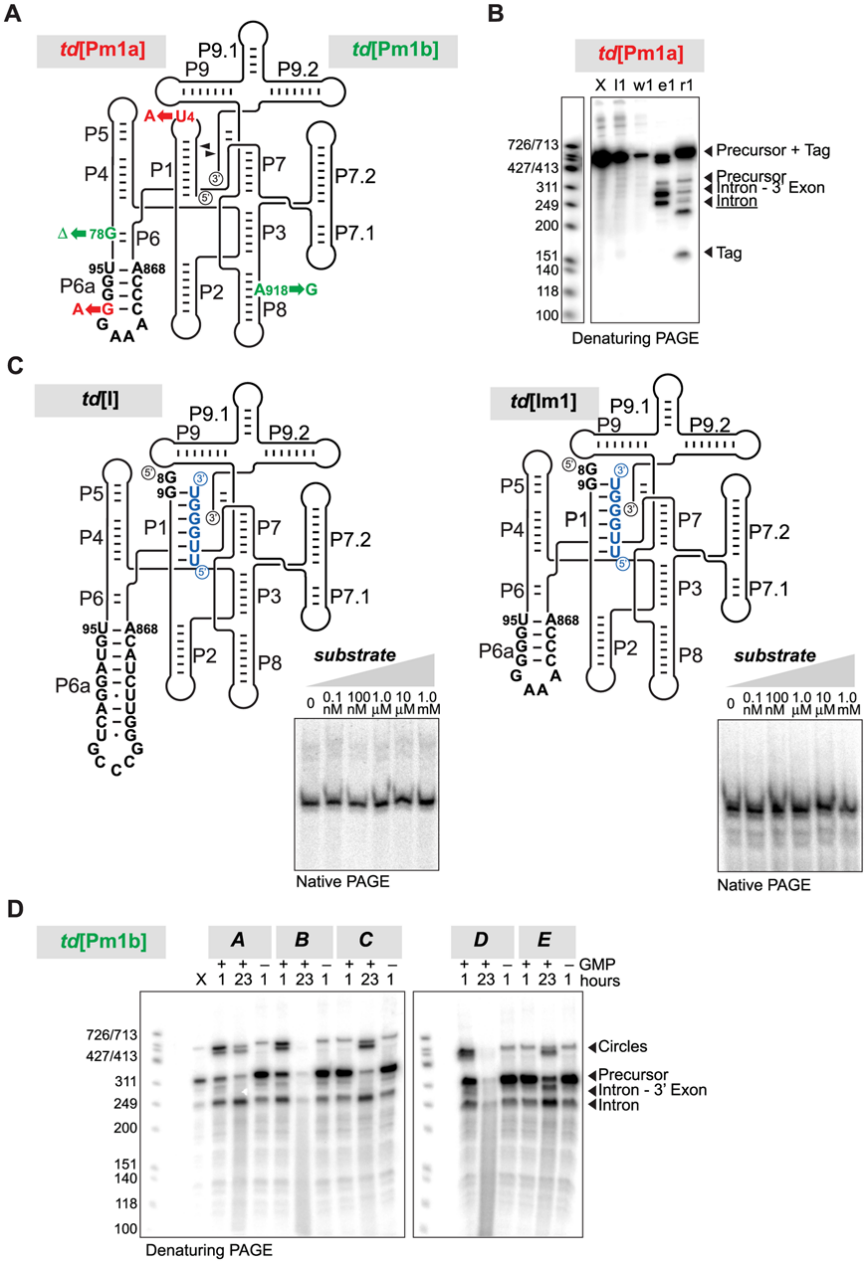
Self-splicing and folding assays of *td* intron variants. (A) Predicted secondary structure of the *td*[Pm1a] and *td*[Pm1b] mutants. The mutations corresponding to each variant are shown in colors (red, *td*[Pm1a]; green, *td*[Pm1b]). (B) Purification of *td*[Pm1a] showing simultaneous self-splicing. (C) Folding of the *td*[I] and *td*[Im] variants in the presence of increasing concentration of the substrate oligonucleotide (sequence shown in blue on the schematized secondary structure diagrams), as visualized by native PAGE. The different sequences of the P6a regions possessed by the two variants are specified on secondary structure diagrams. (D) Self-splicing activity of *td*[Pm1b] under five different buffer conditions (see Methods and [23]).

Of the three RNAs that could not be satisfactorily purified, one had the longest sequence tested (*C.b.*[P]: 683 nt; **Figure 3A**), and two self-spliced or hydrolyzed during the purification, indicating that this method was not suitable for the purification of certain self-splicing introns (*A.s.*[P], **Figure 3A**; *td*[Pm1a], **Figure 4A–B**). In an attempt to inhibit self-splicing and metal-ion mediated hydrolysis during purification, we tested the efficiency of the purification method at conditions that decrease pH (6.5, 7.0 or 7.5), lower the magnesium concentration (2 mM instead of 10 mM), or replace magnesium ions by calcium ions (only the composition of the buffers used for the native purification were modified accordingly). The quality of the purification was assessed for a mutant of the *A.s.*[P] variant transcribed in standard conditions (*A.s.*[Pm1]; **Table 1**). At 2 mM MgCl_2_, the recovered *A.s.*[Pm1] variant was only 75% pure (compared to 90% at 10 mM), because of a poorer yield (20%) at that lower magnesium concentration (**Figure S3**). Hence, that condition was not employed for the purification of other sequences. The other attempts failed to purify the *A.s.*[Pm1] variant (yields<10%, together with severe degradation), in spite of >75% binding to the resin (**Figure S3**).

### Folding and catalytic activity assays

*td* and *C.b.* variants solely containing the intron were annealed in the presence of a substrate oligonucleotide designed to form the P1 helix [20]. Similar complexes were previously crystallized using such strategies [21], [22]. In our case, the sequences of the substrate oligonucleotides were 5′-UUGGGU-3′ for the *td* intron, and either 5′-CGAU-3′ or 5′-CGAdT-3′ for the *C.b.* variants.

**Figure 5:**
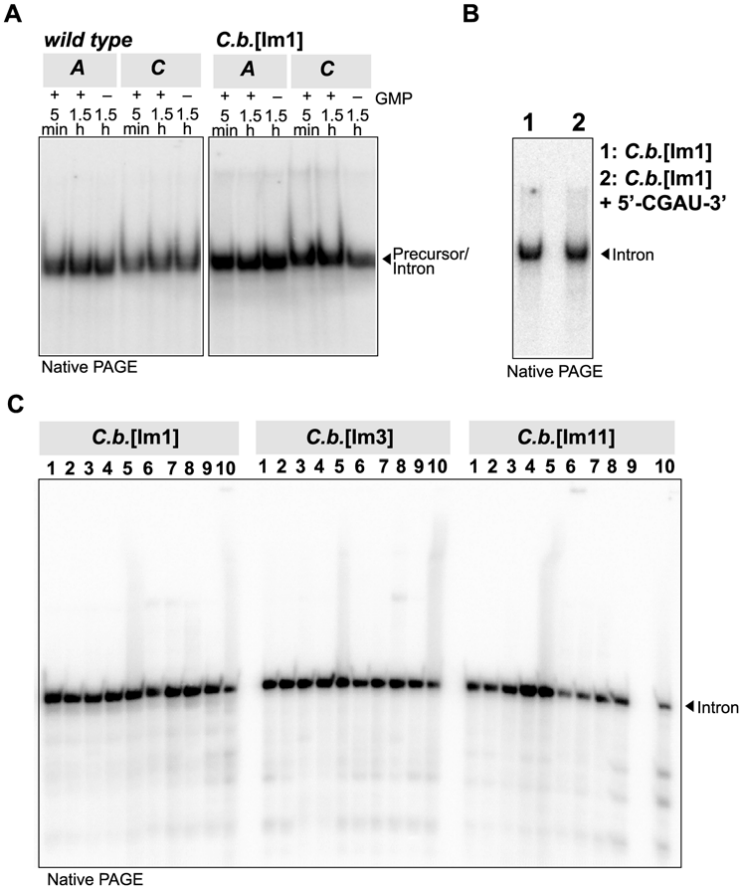
Folding assays of *C. botulinum* intron variants. (A) Folding of the PAGE-purified wild type and the *C.b.*[Im1] introns after incubation for either 5 min or 1.5 h in two buffer conditions, in the presence or absence of 0.1 mM GMP (see Methods and [23]), and visualized by native PAGE. The wild type intron contained an 8 nt-long 5′ exon and a 7-nt long 3′ exon. (B) Comparison of the migration by native PAGE of the PAGE-purified *C.b.*[Im1] variant after incubation for 1.5 h in buffer A (see Methods), either in absence (lane 1) or in presence of 100 µM of a substrate oligonucleotide (lane 2). (C) Folding of three natively purified *C.b.* variants under different annealing protocols, as follows (all conditions in 10 mM Na cacodylate pH 6.5): 1, 2 µM RNA, no annealing protocol; 2, 2 µM RNA, 2.5 µM 5′-CGAdT-3′, no annealing protocol; 3, same as 2 but at 15 mM MgCl_2_, 25 mM NaCl; 4, same as 2 but at 1.0 mM MgCl_2_; 5, same as 1 but at trace [RNA]; 6, same as 1 but incubated for 3 min at 85°C, for 1 min at 25°C, followed by addition of 15 mM MgCl_2_; 7, same as 2 but annealed like 6; 8, same as 3 but incubated for 10 min at 50°C, and for 10 min at 4°C; 9, same as 4 but annealed like 8; 10, same as 5 but annealed like 6. The native gel was run at 15 mM MgCl_2_, 25 mM NaCl.

Prior to crystallization assays, we first tested for folding homogeneity of denaturing PAGE-purified *C.b.* intron variants by native gel electrophoresis, in absence and in presence of the substrate oligonucleotide. A single sharp band was taken to indicate a homogeneously folded molecule. According to this criterion, the PAGE-purified circularly permuted *C.b.*[Im1] RNA was folded similarly to the PAGE-purified wild type molecule (**Figure 5A**), both in absence and in presence of the 5′-CGAU-3′ oligonucleotide (**Figure 5B**). Similarly, native gel experiments showed that the affinity-purified *td*[I] and *td*[Im1] RNAs were folded homogeneously in absence as well as in presence of increasing concentrations of the substrate strand (**Figure 4C**). Finally, as anticipated, comparison of various refolding protocols for three affinity-purified variants (*C.b.*[Im1], *C.b.*[Im3], and *C.b.*[Im11]) indicated that the RNAs in absence and in presence of the 5′-CGAdT-3′ oligonucleotide were folded after native purification, without the need of any additional annealing protocol (compare condition #1 to the other conditions; **Figure 5C**). In fact, some annealing protocols promoted RNA degradation (see condition #10 for the *C.b.*[Im11] variant, corresponding to an incubation for 3 min at 85°C followed by the addition of 15 mM MgCl_2_; **Figure 5C**).

The *B.p.*[P], *D.m.*[P], and *td*[Pm1b] RNAs were assayed for self-splicing activity using conditions recently compiled to test for self-splicing activity in a comprehensive manner [23]. Self-splicing could not be detected for the partially degraded *B.p.*[P] and *D.m.*[P] introns (**Figure S4**). The *td*[Pm1b] variant that contains the A918G mutation and the deletion of G78 was self-splicing, although the second step of the self-splicing reaction was only 50% at best after 1 hour (**Figure 4A,D**).

### Crystallization of a variant of the Clostridium botulinum intron

**Figure 6:**
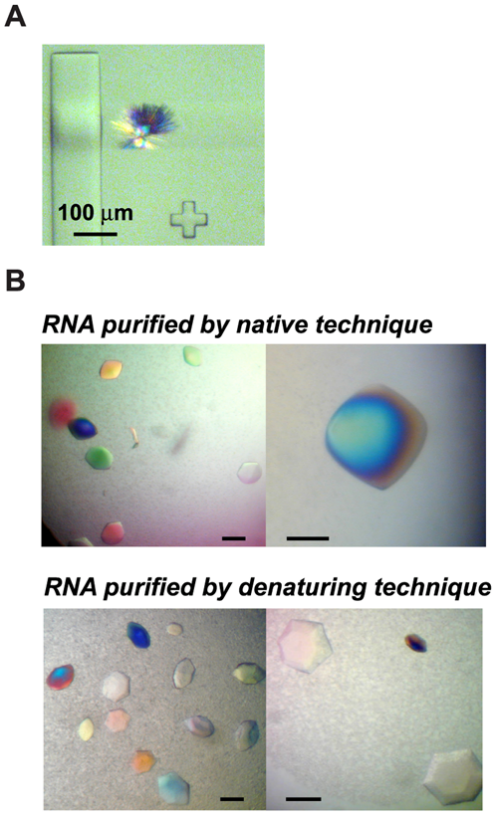
Crystals of the *C.b.*[Im11] variant. (A) Plate cluster observed in a chamber of a micro-chip. (B) Comparison of crystals obtained from natively purified RNA (top) and from RNA obtained by conventional gel purification (bottom). The scale bar corresponds to 100 µm in all panels.

All *td* and *C.b.* variants were assayed by conventional RNA crystallization methods using crystallization screens and either the vapor diffusion technique [24], [25], or the free interface diffusion technique within a micro-chip [26]. Plate clusters of the *C.b.*[Im11] variant were observed after three days at 16°C in the micro-chip, in condition #26 of the PEG/Ion screen (Hampton Research; 10 µM RNA, 12.5 µM 5′-CGAdT-3′, 20% PEG 3350, 200 mM Zn acetate) (**Figure 6A**). Crystals could not be reproduced under these conditions by hanging drop vapor diffusion. However, the same variant crystallized by vapor diffusion after 3 days at 30°C in the following conditions: 20 µM RNA, 25 µM 5′-CGAdT-3′, 10% MPD, 0–80 mM NaCl/KCl, 1 mM Co^3+^ hexamine, 20 mM MgCl_2_, 0–1 mM spermine-HCl, 40 mM Na cacodylate pH 5.5 (drop solution); 25% MPD (well solution). In these conditions, single crystals grew to sizes suitable for diffraction analysis (**Figure 6B**).

The resolution of the *C.b.*[Im11] crystals was ∼10 Å on the CuKα X-ray home source (I/σ = 1.9 and R_merge_ = 32.4% for the 10.6–10.1 Å resolution bin). The space group was trigonal (P3 family), with cell parameters a = 194.1 Å, b = 194.1 Å, c = 415.5 Å, α = β = 90°, γ = 120°. The resolution improved slightly at a synchrotron, to ∼8.0–8.5 Å (**Figure S5A**). Because we were unable to reduce the data collected at the synchrotron, we sought out to obtain crystals using the *C.b.*[Im11] variant purified by a conventional denaturing polyacrylamide gel electrophoresis (PAGE) technique.

The crystals of the PAGE-purified *C.b.*[Im11] variant looked similar to that of their natively-purified counterparts, although they tended to have sharper edges (**Figure 6B**). In spite of this somewhat different morphology, these crystals belonged to the same space group and diffracted in 90% of the cases within the same resolution range as the crystals obtained from natively purified RNA. In the remaining 10% of the cases, we observed a higher resolution at a synchrotron, ∼7.0–7.5 Å (I/σ = 1.6 and R_merge_ = 46.8% for the 8.02–6.96 Å resolution bin; **Figure S5A**). Since about 10 times more crystals of the PAGE-purified RNA were tested than of the natively purified RNA, it cannot be ruled out that similar resolutions could have been achieved with crystals of the natively purified RNA. It is also possible that original crystals of the natively-purified RNA were grown from a solution that contained partially degraded RNA. In fact, in order to preserve the native folding of this intron, we initially stored the RNA at 4°C in the presence of 5 mM MgCl_2_ (in 10 mM Na cacodylate pH 5.0). We later found out that storage of the RNA at 4°C even in 1 mM MgCl_2_ was inducing severe degradation after a few weeks (**Figure S6**). We subsequently stored the RNA at −20°C in absence or in presence of 1 mM MgCl_2_, at pH 5.0, which did not promote RNA degradation (**Figure S6**) nor altered the folding. Finally, spherulites and urchins were obtained in similar conditions for two other variants purified by PAGE, *C.b.*[Im3] and *C.b.*[Im8] (**Figure S5B**; the natively purified *C.b.*[Im3] and *C.b.*[Im8] variants had not been extensively assayed for crystallization). In any case, attempts at annealing crystals to increase resolution [27], [28] were unsuccessful. Furthermore, all crystals tested suffered from severe radiation damage, regardless of the RNA purification method and of the cryoprotecting condition, leading to rapid loss in resolution during data collection.

## Discussion

23 group I intron variants ranging in length from 246 to 341 nt were transcribed and subjected to purification using a non-denaturing affinity purification method. The original protocol [3] was adjusted to allow purification of small-scale transcription reactions. Using this technique, the overall time required for the production of six samples in parallel was less than two days. Besides the advantages of the native purification previously emphasized [3], two factors were critical to attaining such a fast time scale. First, the sequence of the DNA template was split into five primers that could be amplified altogether in one round of PCR using a high-fidelity DNA polymerase. Although successful amplification of 6–8 overlapping primers was achieved, these products tended to contain a higher frequency of random mutations. Noteworthy, we were unable to amplify the only sequence in our screen that was made of 10 overlapping primers. These observations expand on previous reports of PCR synthesis from overlapping oligonucleotides of 100–150 nt long sequences [29]. Second, a 3′ overhang of one nucleotide introduced in the series of the *C.b.*[Im6–12] variants enabled optimal cleavage by HDV. The sequence of this overhang did not seem to be critical, as variants ending with a single A (e.g. *A.s.*[Pm1], *C.b.*[Im6] and *C.b.*[Im11]; **Figures 3B** **,** **S2** **and** **S3**), a single U (e.g. *C.b.*[Im10]; **Figure S2**), a single C (e.g. *C.b.*[Im5]; **Figure 3B**) were efficiently cleaved off the resin by HDV (yield>50%; **Table 1**). However, the overhang of *C.b.*[Im4] containing two cytidines likely interfered with folding of the HDV ribozyme, resulting in poor cleavage (**Figure 3B** **;** **Table 1**). Overall, when this fast preparation of pure RNA was coupled to crystallization assays for which we used the free interface diffusion system, the time span between RNA molecule design and in-chip crystal observation was under two weeks. This represents a faster timeline than when applying conventional methods [**25**], particularly when working with RNAs of that size.

Although it was not completely clear from our data whether the quality of the natively-purified and the PAGE-purified *C.b.*[Im11] RNAs were the same, both RNAs crystallized under the same conditions and the crystals looked similar. This encouraging observation suggested that this purification method would be suitable for a fast screening of several crystallization variants during the initial screening steps, even for sequences about 300 nt long. Furthermore, if results of such a screening ended up being ambiguous, the PAGE-purification of some of the most promising variants could always be chosen as part of the optimization strategy. Although RNAs of this length have not yet been tested with the updated version of the purification scheme [8], the purification efficiency should be similar. In particular, we expect that the elimination of the incubation step in the presence of imidazole to make for a critical advance in purifying long RNA molecules, since it should limit RNA degradation. Finally, special care should be given to the storage condition of the natively-purified molecules, so that the native structure would be preserved while the metal ion-mediated degradation of the RNA would be minimized.

Even when following these recommendations, this purification method may not be suitable for all RNAs, particularly autocatalytic molecules. For example, some precursor RNAs (e.g. the *A.s.*[P] and the *td*[Pm1a] variants; **Figures 3A** **and** **4A**) hydrolyzed or self-spliced over the course of the purification process, which occurs at 10 mM MgCl_2_ and may contain some traces of GTP employed during transcription (**Figure 1B**). Performing the purification at 2 mM MgCl_2_ may be sufficient to reduce self-splicing for some introns, although such a lower magnesium concentration may additionally result in poorer yields of pure RNA (**Figure S3**). Fortunately, not all introns self-cleaved during the purification procedure (e.g. the *td*[Pm1b] variant; **Figure 4D**), likely because a condition that favors hydrolysis or self-splicing for one intron may not have the same effect on another intron [23]. Consequently, the purification system needs to be tested on a case-by-case basis. Because of the rapid timeline by which results are obtained, reverting to a conventional purification method would happen in a timely manner.

Finally, the coupling of the PCR strategy employed here and of the native purification method could help engineer random mutations in the specific regions of RNA molecules about 300 nt long (i.e. by including such areas within the overlap regions). Such mutants may lead to improvement in crystallization, as mutation of one nucleotide often strongly affects resolution [30]. In our case, such mutations resulted in *td* mutants that unexpectedly retained some activity. The *td*[Pm1a] RNA self-spliced over the course of the purification, indicating that the two base substitutions in the L1 loop and in the P6a stem did not hamper activity (**Figure 4A–B**). Self-splicing of the *td*[Pm1b] RNA was initially unforeseen, as the deletion of G78 normally paired to C865 at the first position of the P6 helix (P6-bp1) likely disrupted the corresponding base triple involving P6 and the J3/4 junction in all group I introns [31]. However, mutation of G to C at position 78 [32] and of the adjacent C to U at position 79 [33] had been shown to have a similar effect on splicing. Similarly to the partial suppression of the C79U mutation observed upon addition of the CYT-18 protein [34], [35], it could be worth investigating whether the activity of the *td* intron deprived of G78 could be rescued by the CYT-18 protein or by the StpA protein, which also binds to this location [36].

### Concluding remarks

While developing a second generation of the affinity purification method [8], we showed that by using the first generation method we successfully purified RNA molecules as long as ∼341 nt. These RNAs retained a folded structure that enabled them to be active in self-splicing assays. One variant of the *Clostridium botulinum* intron gave crystals of a similar quality than crystals obtained using a conventional denaturing purification technique, although the time span to obtain the RNA was significantly reduced. Together, the first and the second generation of the purification techniques may constitute unique tools for the preparation of co-transcriptionally folded RNAs of a large size.

## Materials and Methods

### Intron selection and construct design

Group I introns were selected based on their length (ranging from 167 nt to 393 nt), their exon context (three tRNA^Leu^ introns, one ribosomal intron, one tmRNA intron, and one intron in a protein coding sequence), and other properties relating to the purpose of this study. For example, three introns (*A.s.*, *td*, *C.b.*) had been previously shown to be self-splicing [11], [13], [23] and were used to test the efficiency of the purification method. Constructs were designed that incorporated the intron embedded in both exons, either at their natural length (for tRNA and tmRNA exons), or shortened to tRNA-like lengths (for *td* and *S.d.* exons). Additional variants were synthesized to incorporate the intron only, for *td* (two variants missing the first seven nucleotides [20] and containing shortened P6a regions [37]) and *C.b.* (12 circularly permuted variants possessing diverse P6a regions).

Constructs were designed to incorporate the following sequence: six random nucleotides, a restriction site for cloning at the 5′ end (EcoRI), the T7 polymerase promoter 5′-TAATACGACTCACTATA-3′, three guanosine residues, the nucleotides corresponding to the 5′ exon (when appropriate; Table 1), the intron, the nucleotides corresponding to the 3′ exon (when appropriate; Table 1), a restriction site for cloning and/or linearization at the 3′ end (BbsI or NcoI), six random nucleotides. The construct sequence was split into five to ten 65–85-nucleotide long DNA primers that were overlapping by 15–20 nucleotides (note: approximately half of these primers would hereby contain the sequence of the complementary strand) (**Table S1**). Polymerase chain reactions (PCR) from overlapping DNA primers spanning an entire gene thus offered an alternative to cloning genomic DNA [16], [17].

### Multi-round PCR strategy

For the first round of PCR, constructs were amplified in 50 µL-reaction mixes containing: 0.4 pmoles of each overlapping primer (∼10 ng); 200–250 µΜ each dNTP; 1.0 mM MgCl_2_; either 0.5 U Taq DNA polymerase (Roche #11 146 165 001), or 0.5 U Pwo DNA polymerase (Inno-Train Diagnostik GbmH #GX02010), or 2.5 U Herculase DNA polymerase (Stratagene #600262); 1X reaction buffer supplied by the manufacturer. PCR were performed in a Robocycler Gradient 96 apparatus (Stratagene) using the following protocol: 4 min at 94°C (1 cycle); 55 s at 94°C, 55 s at 50–65°C, 1.5 min at 72°C (30–35 cycles); 10 min at 72°C (1 cycle). When only one round of PCR was necessary, these conditions were employed, in the presence of the generic primers described below for the second round.

When a second round of PCR was necessary, PCR products from the first round were combined as follows in 50 µL-reaction mixes: 20 µL of each of the two sets of amplified portions from the first round necessary to obtain the complete sequence of the construct (e.g. for the *td*[P] construct: 20 µL of the amplified primers 1−4+20 µL of the amplified primers 3–7); 50 pmoles of each of the two generic primers (containing the first 25–30 nucleotides of the first and the last overlapping primer); 200–250 µΜ each dNTP; 1.0 mM MgCl_2_; either 0.5 U Taq DNA polymerase (Roche #11 146 165 001), or 0.5 U Pwo DNA polymerase (Inno-Train Diagnostik GbmH #GX02010), or 2.5 U Herculase DNA polymerase (Stratagene #600262); 1X reaction buffer supplied by the manufacturer. PCR were performed in a Robocycler Gradient 96 apparatus (Stratagene) using the same cycles as for round #1. The *A.s.*[Pm1], *D.m.*[P] and *B.p.*[P] variants were subsequently amplified for a third round in presence of a primer replacing the BbsI restriction site by a NcoI/NgoMIV site (see below; ‘As-Ngo’ and ‘Dm/Bp-Ngo’ primers, **Table S1**).

All PCR products were monitored by electrophoresis on 1% or 1.5% agarose gels run using 1X TAE buffer (40 mM Tris-acetate pH 8.0; 1.0 mM EDTA). Gels were stained using ethidium bromide (Sigma #46067-50ML-F) and visualized using a UV-light transilluminator.

### Cloning procedure and transformation

The PCR amplified products were purified using the QIAquick PCR purification kit (Qiagen #28104), and digested using the appropriate restriction enzymes (EcoRI, New England Biolabs (NEB) #R0101L; and NcoI, NEB #R0193L—incomplete digestion products were typically obtained using BbsI, NEB #R0539L, which made us avoid using this enzyme). The digested products were then purified by agarose gel electrophoresis, and eluted in a final volume of 30 µL using the QIAquick gel extraction kit (Qiagen #28706). Ligation occurred for 35 min at room temperature (∼23–25°C) in a solution containing: 0.2 µg/µL pRAV12 vector [3]; 50–100 ng/µL PCR product; 400 U T4 DNA ligase (NEB #M0202S); 1X reaction buffer supplied by the manufacturer. The pRAV12 vector had been previously digested using the EcoRI and NcoI or NgoMIV restriction enzymes and dephosphorylated using 1.0 U calf intestine alkaline phosphatase (Roche #713023) for 30 min at 37°C followed by 20 min at 70°C. 1.0 µL of the ligation reaction mix was used to transform 10 µL of Solopack Gold Supercompetent cells (Stratagene #230350). Transformation was performed using the following protocol: 20 min at 4°C; 1 min at 54°C; addition of LB (-Amp) broth; incubation on LB (+Amp) plates for 16–20 h at 37°C. Alternatively, 1.0 µL of the ligation reaction mix was used to transform 40 µL of XL1-Blue electroporation-competent cells (Stratagene #200228). Electroporation was performed according to the manufacturer's protocol, using 0.1-cm gap cuvettes (Bio-Rad #165-2083EDU) and a Bio-Rad Gene Pulser set to a 200 Ω resistance, and a 25 µF capacity. In both cases, plasmids possessing the expected sequence were purified using the QIAprep spin miniprep kit (Qiagen #27106) from 3–5 mL LB cultures grown for 16–20 h at 37°C.

The *C.b.*[P] and the *A.s.*[P] constructs were independently amplified from plasmids containing the intron and the complete exons (for *C.b.*[P]: p237AK, gift from K. Williams; for *A.s.*[P]: pAtRNA-1, [38]), using the generic primers and 2.5 U Herculase DNA polymerase (similar strategy as described for the second round of PCR above). The *C.b.*[Im1] variant was amplified from the p237AK plasmid using a two-step PCR strategy that would circularly permute the 5′ and 3′ ends into the P6a region (**Figure S7**). Each PCR step was performed under the reaction conditions described above. The PCR products of the first step were diluted by 10-fold and 1 µL of this dilution was used as a template into a final 50 µL-reaction mix for the second step. The various 5′ and 3′ ends of the *C.b.*[Im2–12] variants (**Figure 2**) were added by PCR using *C.b.*[Im1] as a template and the primers reported in **Table S1**. The *C.b.*[P], *A.s.*[P] and *C.b.*[Im1–12] constructs were ligated into pRAV12 and expressed according to the procedure described above.

### In vitro transcription of body-labeled RNAs

Plasmids were linearized using the BamHI restriction enzyme (NEB #R0136L) and were transcribed for 1 h at 37°C in 25 µL-reaction mixes containing: 1.0–4.0 µg/µL linearized plasmid; 50 µCi α-^32^P ATP (PerkinElmer); 4.0 mM each rNTP; 12.0 mM MgCl_2_; 2.0 mM spermidine-HCl; 10 mM DTT; 0.1% Triton X-100; 4.0 ng/µL inorganic pyrophosphatase (Sigma #I1891-100UN); 40 U RNasin Plus RNase inhibitor (Promega #N261B); 2 µL T7 RNA polymerase (prepared in house); 50 mM Tris-HCl pH 7.5. The final radioactivity of the samples was measured by the number of counts per minute (cpm) for 1.0 µL of the RNA solution diluted into 3.0 mL ScintiSafe Econo 1 solution (Fisher Scientific #SX20-5), obtained using a scintillation counter (Beckman #LS3801).

### In vitro transcription of unlabeled RNAs

Linearized plasmids each containing one of the *C.b.*[Im1–12], *td*[I], or *td*[Im1] variants were transcribed in a similar reaction solution, but for 2 h at 37°C and in 1.5 mL–10.0 mL reaction mixes. Concentrations of the purified products were determined from the A_260_ and calculated extinction coefficients based on the nucleotide sequence (using for example a resource such as the following: http://www.basic.northwestern.edu/biotools/oligocalc.html). The RNAs were concentrated to ∼500 µM using a centrifugal filter unit with a 10,000 MWCO (Millipore, UFC801008), and stored at 4°C or at −20°C in a buffer containing either 50 mM Na HEPES pH 7.5, 25 mM NaCl, 15 mM MgCl_2_, or 10 mM Na cacodylate pH 5.0–6.5, 10 mM NaCl and 0–5 mM MgCl_2_. The *C.b.* RNAs were used in crystallization assays without prior refolding, while the *td* variants were annealed for 10 min at 37°C prior to being utilized in crystallization assays.

### RNA preparation by a denaturing technique

The gel-purified *C.b.*[Im1–12] variants used as a control for the crystallization and X-ray diffraction experiments were transcribed by run-off transcription from a plasmid linearized using the FokI enzyme (NEB #R0109S), as described above, but for 4 h at 37°C and in 10 mL-reaction mixes. The RNAs were ethanol precipitated for 16 h at −20°C in 70% EtOH, 100 mM Na acetate pH 5.3, centrifugated for 35 min at 13,500 g, dried for 10 min under vacuum (SpeedVac), and purified by denaturing gel electrophoresis (6% acrylamide/bisacrylamide [29∶1]; 8.0 M urea; 1X TBE). The gel slice corresponding to the RNA was crushed and soaked for 2 h at 4°C in 35 mL of an elution buffer containing: 1.0 mM EDTA; 10 mM Tris-HCl pH 7.5. The solution was filtered through a 0.2 mm filter, and concentrated to ∼200 µM using a centrifugal filter unit with a 30,000 MWCO (Millipore #UFC903096), and stored at 4°C or −20°C in a buffer containing 10 mM Na cacodylate pH 6.5, 10 mM NaCl and 5 mM MgCl_2_. The RNAs were used in crystallization assays without prior annealing.

A similar procedure was applied to a 25 µL-run off transcription reaction of the radiolabeled *A.s.*[Pm1] RNA that was deprived of the affinity tag. Here, the gel slice corresponding to the *A.s.*[Pm1] RNA was crushed and soaked for 20 h (with one buffer exchange after 2 h) at 4°C in 800 µL of an elution buffer containing: 1.0 mM EDTA; 250 mM NaCl; 10 mM Tris-HCl pH 7.5. Precipitation and filtration were performed as described in the previous paragraph, and the RNA was stored at −20°C in water. The final radioactivity of the RNA was measured as described in the section entitled: “*In vitro transcription of body-labeled RNAs*”.

### Preparation of the TmaM affinity matrix

The *TmaM* protein was prepared and coupled to the activated support as described [3] (**Figure S8**). Over the course of this preparation, similar problems to that later reported were observed, including for example the precipitation of the protein after its concentration [8]. Attempts to refold the protein in 8.0 M urea at various temperatures (37°C, 50°C, 70°C) were unsuccessful.

### Nondenaturing purification of the transcripts

The body-labeled transcription products were purified by nondenaturing purification as described [3], with the modifications mentioned in Figure 2. G-25 Sephadex quick spin columns (Roche #1 273 990) and spin columns (Qiagen #79523 or Pierce #29924) were employed during the purification. Buffers used were as follows [3]: wash buffer (**WB**), 10 mM MgCl_2_, 250 mM NaCl, 25 mM Tris-HCl pH 8.0; cleavage buffer (**CB**), 10 mM MgCl_2_, 250 mM NaCl, 200 mM imidazole, 25 mM Tris-HCl pH 8.0; regeneration buffer (**RB**), 1.0 M LiCl, 174 mM glacial acetic acid, 25 mM Na_2_EDTA; storage buffer (**SB**), WB containing 0.1% Na azide.

The unlabeled transcription products of the *C.b.*[Im1–12] variants were purified without modification of the published protocol (except for adjustments to the corresponding transcription reaction volumes) [3].

Products from the purification were analyzed by electrophoresis on denaturing gels (6% acrylamide/bisacrylamide [19∶1]; 7.0 M urea; 1X TBE: 100 mM Tris-base, 83 mM boric acid, 1.0 mM EDTA). Care was taken to load gels with volumes of samples proportional to the total volume of each fraction (except for the *C.b.*[Im1–12] variants). The gels were either stained using SYBR Green II RNA gel stain (Invitrogen, #S-7568; only for the *C.b.*[Im1–12] variants), or dried under vacuum and placed for 18–30 h in phosphorimager screens (Molecular Dynamics). Both the stained gels and the screens were scanned using a phosphorimager (Amersham Bioscience/GE) and the gel images were analyzed using ImageQuant TL v. 2005 (Amersham Bioscience/GE).

An approximate yield of cleavage from the resin was estimated by calculating the average between y_1_  =  [(N_1_/26)/((N_1_/26) + (N_2_/n_A2_)] × 100 and y_2_  =  [(N_3_/n_A3_)/((N_3_/n_A3_) + (N_2_/n_A2_)] x 100, where:

N_1_ = number of counts in the band corresponding to the tag removed from the support during the regeneration steps (found in lanes “r1–r5” on Fig. 3, S2 and S3);

N_2_ = number of counts in the band corresponding to the uncleaved RNA removed from the support during the regeneration steps (found in lanes “r1–r5” on Fig. 3, S2 and S3);

N_3_ = number of counts in the band corresponding to the RNA cleaved off from the support during the elution steps (found in lanes “e1–e5” and/or “pool A/B” on Fig. 3, S2 and S3);

n_A2_ = number of adenines in the precursor/intron+tag RNA (the tag contains 26 adenines);

n_A3_ = number of adenines in the precursor/intron RNA (110, *A.s.*[P] and *A.s.*[Pm1]; 95, *D.m.*[P]; 107, *B.p.*[P]; 78, *td*[Pm1]; 98, *C.b.*[Im11]).

Purity was estimated by the ratio [(N_3_/(N_3_+N_total_)] × 100, where N_3_ is as described above, and:

N_total_ = total number of counts other than N_3_ (found in lanes “e1–e5” and/or “pool A/B” on Fig. 3, S2 and S3);

All the corresponding lanes shown in Figs. 3, S2 and S3 were used for quantification. In the cases shown in Figs. 2 and S3, the volumes loaded on the gels were proportional to the total fraction volumes. In the gels shown on Fig. S2, the number of counts were normalized to take into account differences in the ratios of volume loaded on the gel/total fraction volume. All elution and regeneration fractions were used to calculate the yields of the variants displayed on **Figs. 3B** **,** **S2** **,** **S3**. Noteworthy, the yields for *A.s.*[P], *D.m.*[P], *B.p.*[P], and *td*[Pm1] were calculated using the amounts of cleaved off and uncleaved RNAs present in lanes e1 and r1 only (Fig. 3A). Based on the yields for *A.s.*[Pm1] and the *C.b.* variants it was assumed that >70% of the product is recovered during the first elution step and >70% of the uncleaved RNA is removed from the resin during the first regeneration step (**Table 1** **;** **Fig. 3A**).

### Folding assays

Approximately 2,000 cpm of the body-labeled wild-type *C. botulinum* intron and of the *C.b.*[Im1] variant purified by denaturing PAGE were annealed for 3 min at 50°C followed by 10 min at 32°C in 25 mM Na HEPES pH 7.5 [38], before addition of solution **A** or **C** (see Self-splicing assays section below) and further incubation for 5 min or 1.5 h at 32°C. A sample of the *C.b.*[Im1] variant was also similarly incubated in the presence of 100 µM 5′-CGAU-3′.

Approximately 20,000 cpm of the *C.b.*[Im1], *C.b.*[Im3] and *C.b.*[Im11] variants natively purified were incubated according to different annealing protocols, as described in the legend to Figure 5 (all conditions in 10 mM Na cacodylate pH 6.5).

Approximately 5,000 cpm of the body-labeled *td*[I] and *td*[Im] RNAs were annealed for 10 min at 50°C followed by 10 min at 25°C, in a solution containing 5.0 mM MgCl_2_, and 25 mM Na HEPES pH 7.5. These samples were added to various concentrations (0.1 nM, 100 nM, 1.0 µM, 10 µM, 0.1 mM) of an RNA hexamer (sequence: 5′-UUGGGU-3′) corresponding to the end of the 5′ exon and necessary for the formation of the P1 helix [20]. Samples were incubated for 10 min at 37°C, added to 50% glycerol, and loaded on native polyacrylamide gels (6% acrylamide/bisacrylamide [19∶1]; 1X TBE; 10 mM MgCl_2_; run at 4°C). The gels were dried and analyzed as described above.

### Self-splicing assays

The *D.m.*[P], *B.p.*[P] and *td*[Pm1b] RNAs were tested for self-splicing activity under a set of conditions previously reported to efficiently assay self-splicing of group I introns [23]. Self-splicing reactions proceeded for 1 h or 24 h at various temperatures (see below) in 10 µL-reaction mixes containing 50 nM RNA (annealed for 3 min at 50°C, then for 10 min at 32°C in 25 mM Na HEPES pH 7.5 [38]), 0.1 mM GMP, in one of the following buffers: **A**, 15 mM MgCl_2_, 25 mM NaCl, 25 mM Na HEPES pH 7.5 (incubation at 32°C); **B**, 15 mM MgCl_2_, 25 mM NaCl, 25 mM Na HEPES pH 7.5 (incubation at 42°C); **C**, 15 mM MgCl_2_, 1.0 M NaCl, 25 mM Na HEPES pH 7.5 (incubation at 32°C); **D**, 200 mM MgCl_2_, 25 mM NaCl, 25 mM Na HEPES pH 7.5 (incubation at 32°C); **E**, 10 mM Mg(OAc)_2_, 0.4 M NaOAc, 25 mM Na HEPES pH 7.5 (incubation at 32°C). Reactions were stopped by the addition of a solution containing: 500 mM EDTA; 7.0 M urea; 0.02% bromophenol blue; 0.02% xylene cyanol; 1X TBE (100 mM Tris-base; 83 mM boric acid; 1.0 mM EDTA). Products were analyzed by denaturing polyacrylamide gel electrophoresis (PAGE) as described above.

### Crystallization assays

The native-affinity purified *C.b.* and *td* variants were diluted to 5–60 µM in a solution containing the following components: substrate oligonucleotide (1∶1.25, RNA:oligonucleotide ratio), 0–80 mM NaCl or KCl, 10–20 mM MgCl_2_, 40 mM Na cacodylate pH 5.5 or pH 6.0. Hanging drops were set up in crystallization plates by mixing 0.5–1.5 µL of the RNA solution and 0.5–1.5 µL of a crystallization solution (0.5–1 mL in the well) from the following screens: Natrix (Hampton Research (HR) #HR2-116); PEG/Ion Screen (HR #HR2-126); the MPD Suite (Qiagen #130706); a modified version of the Nucleic Acid Mini-Screen (HR #HR2-118), containing 2.5–15% MPD in the drop (18–35% MPD in the well), 1 mM Co^3+^ hexamine or 1 mM spermine; a screen prepared in house, containing as precipitant either 5–30% 1,6-hexanediol, or 5–15% isopropanol, or 1.4–1.8 M (Li)_2_SO_4_, or 1.6–2.2 M (NH_4_)_2_SO_4_, and 2–10 mM Mg(OAc)_2_, 1 mM spermine, 2 mM Co^3+^ hexamine, 50 mM Na cacodylate pH 6.5. Free interface diffusion experiments were set up in Topaz chips at the 4.96 format (Fluidigm, www.fluidigm.com) using 1 µL of RNA solution and 10 µL of crystallization solution per well according to manufacturer's instructions. The chips were incubated at 16°C.

### X-ray diffraction data collection

The first crystals obtained for the *C.b.*[Im11] variant were cryoprotected for 10 min in 60% MPD and flash-frozen in liquid nitrogen. Data were collected on an R-AXIS IV++instrument with CuKα X-ray radiation using 1° oscillation angle and 30–45 min exposures. A 20° wedge of data collected with 45 min exposure time per frame was reduced using d*TREK (51% completeness).

Additional crystals of the natively-purified and *C.b.*[Im11] variant were flash-frozen in liquid nitrogen directly from the hanging drop, or were first cryoprotected for 5–20 min in various cryoprotecting solutions (20–60% MPD; 5–20% isopropanol; 10–20% ethanol; 10–30% MPD together with 10% glycerol or ethylene glycol). Data were collected at the Howard Hughes Medical Institute 8.2.1 beamline at the Advance Light Source (Berkeley, CA), using 0.5° oscillation range and 15 s exposures. A 20° wedge of data was reduced using MOSFLM version 7.0.4 [39] (41% completeness).

## Supporting Information

Table S1 Generic and overlapping primers used in this study.
(0.03 MB XLS)

Figure S1 PCR synthesis using overlapping primers. Products are visualized on agarose gels stained using ethidium bromide. Expected products are marked by a black triangle. Labels above each lane indicate the number of overlapping primers (C: control reactions containing only the generic primers). Labels below each lane refer to the DNA polymerase used during PCR (T: Taq; P: Pwo; H: Herculase). A star symbol indicates when 5.0 U instead of 2.5 U Herculase were used. The annealing temperature is shown in yellow on the top right corner of each gel.
(2.13 MB TIF)

Figure S2 Purification assays of the *C.b.*[Im6], *C.b.*[Im7], *C.b.*[Im10], *C.b.*[Im11] and *C.b.*[Im12] variants. These variants were purified using the large-scale purification method (purification of 10-mL transcription reaction mixes). The assays were visualized on 6% denaturing PAGE, and stained using SYBR Green II. Labels above each lane refer to purification steps detailed in Figures 1B and 3.
(0.97 MB TIF)

Figure S3 Purification assays of the *A.s.*[Pm1] variant at lower MgCl_2_ concentration, using CaCl_2_ in place of MgCl_2_, and at pH 6.5–8.0. The results are visualized by denaturing PAGE as in Figure 3; lanes are labeled according to the purification steps specified in Figure 1B.
(4.10 MB TIF)

Figure S4 Self-splicing assays of the *D.m.*[P] and *B.p.*[P] variants. The self-splicing activity was tested under five different buffer conditions (only three shown; see Methods and [21]).
(0.33 MB TIF)

Figure S5 Diffraction patterns of *C.b.*[Im11] variants and crystalline materials obtained for the *C.b.*[Im3] and *C.b.*[Im8] variants. (A) Diffraction patterns of crystals grown using either the natively purified RNA (left), or the RNA purified by denaturing PAGE (right). The 7.0–8.5 angstrom resolution typically obtained for these crystals is indicated in red. (B) Spherulites (left) and urchins (right) obtained with the PAGE-purified *C.b.*[Im3] and *C.b.*[Im8] variants, respectively.
(3.66 MB TIF)

Figure S6 Analysis of the *C.b.*[Im11] RNA stock after eight weeks of storage in various conditions. The RNA was in 10 mM Na cacodylate pH 5.0 in all cases. Approximately 10 pmoles of RNA were loaded in each lane. The assays were visualized on 6% denaturing PAGE, and stained using SYBR Green II.
(0.29 MB TIF)

Figure S7 A two-step PCR strategy to circularly permute the 5′ and 3′ ends of the *C.b.* intron into the P6a region. During the first PCR step, the wild-type *C.b.* intron (colored from the wild-type 5′ end to the P6a helix (red), and from the P6a helix to the wild-type 3′ end (brown)) is used as a template for two independent PCR rounds that contain different sets of primers. During the second PCR step, the product of each of these reactions is combined to suitable primers from step #1 in order to amplify the expected circularly permuted *C.b.* intron. The incorporated T7 promoter and restriction sites suitable for cloning are colored as in Figure 1.
(0.53 MB TIF)

Figure S8 Expression and purification of the *Tma*M-domain protein. (A) Cells prior and post-induction with 1.0 mM IPTG (ten 750-mL cultures were grown for 12 h at 31°C in LB medium). (B) Supernatent fraction and pellets of three cell lysates. (C) Fraction of protein eluted from Ni^2+^-affinity column and after TEV cleavage (three fractions were cleaved for 16 h at 25°C by a 1∶100 ratio (by mass) of TEV protease, in 10 mM DTT). (D) Chromatogram after purification of the *Tma*M-domain on the SP-Sepharose column (12 fractions were collected, as indicated in green). The protein eluted around 0.55 M NaCl in 10 mM MES pH 6.0. (E) Corresponding elution fractions. Fractions #8 and 9 were pooled, dialysed and coupled to the activated support (Affigel-10; Bio-Rad #153-6046). Gels shown in panels A, B, C, and E were from SDS-PAGE using 4–12% or 12% acrylamide (as specified under each gel), and were stained using SimplyBlue SafeStain (Invitrogen #LC6060); ladders: SeeBlue Plus 2 ((A)–(E); Invitrogen #LC5925), BenchMark Pre-stained ((E) only; Invitrogen #10748-010).
(3.29 MB TIF)
