# Supplementary figures and images for: Preparation of Group I Introns for Biochemical Studies and Crystallization Assays by Native Affinity Purification
Source: PLoS One. 2009 Aug 27;4(8):e6740. doi: 10.1371/journal.pone.0006740 (PMC2729099; doi:10.1371/journal.pone.0006740)

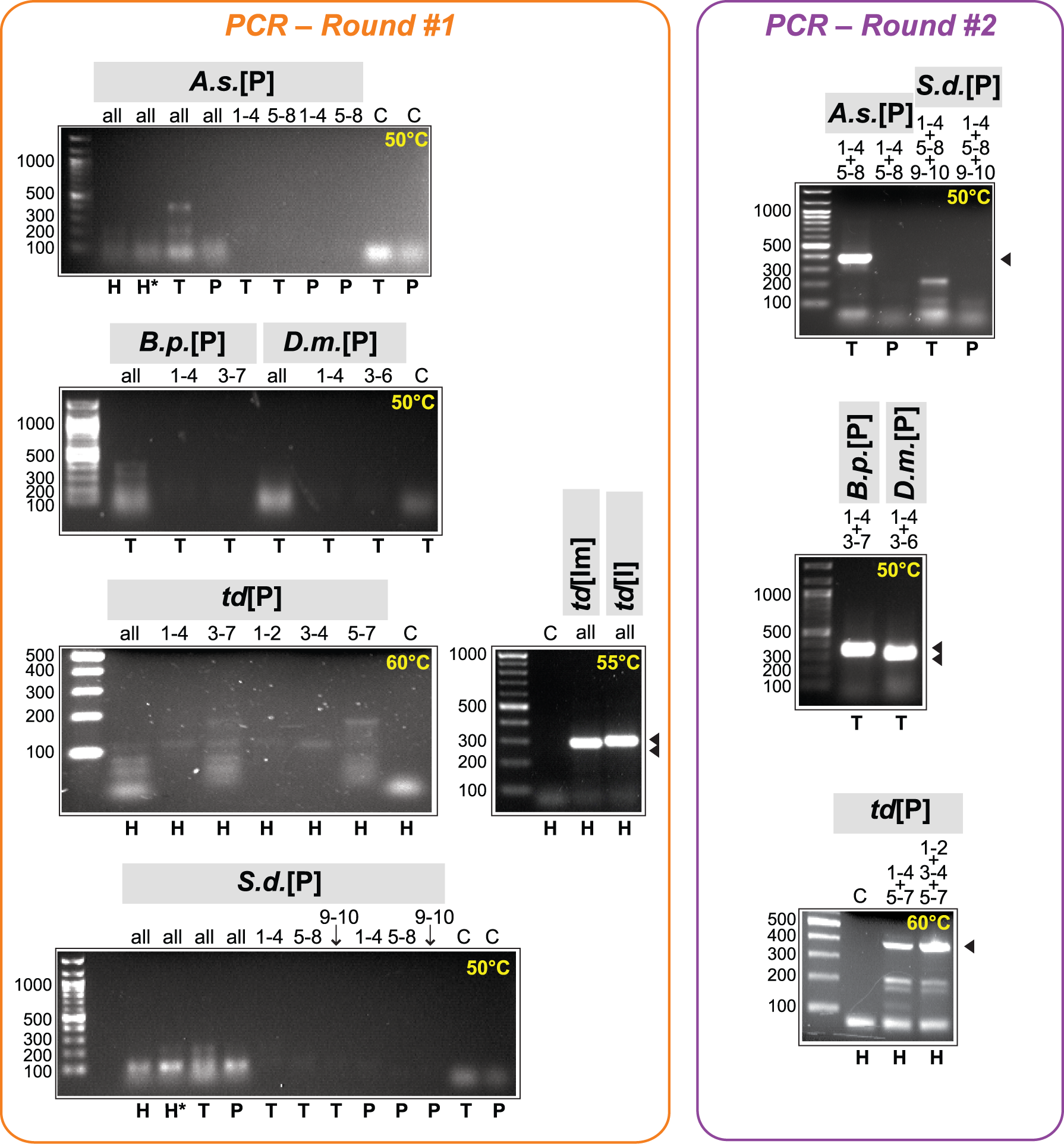

Supplement: Figure S1 — PCR synthesis using overlapping primers. Products are visualized on agarose gels stained using ethidium bromide. Expected products are marked by a black triangle. Labels above each lane indicate the number of overlapping primers (C: control reactions containing only the generic primers). Labels below each lane refer to the DNA polymerase used during PCR (T: Taq; P: Pwo; H: Herculase). A star symbol indicates when 5.0 U instead of 2.5 U Herculase were used. The annealing temperature is shown in yellow on the top right corner of each gel. (2.13 MB TIF) [file pone.0006740.s002.tif]

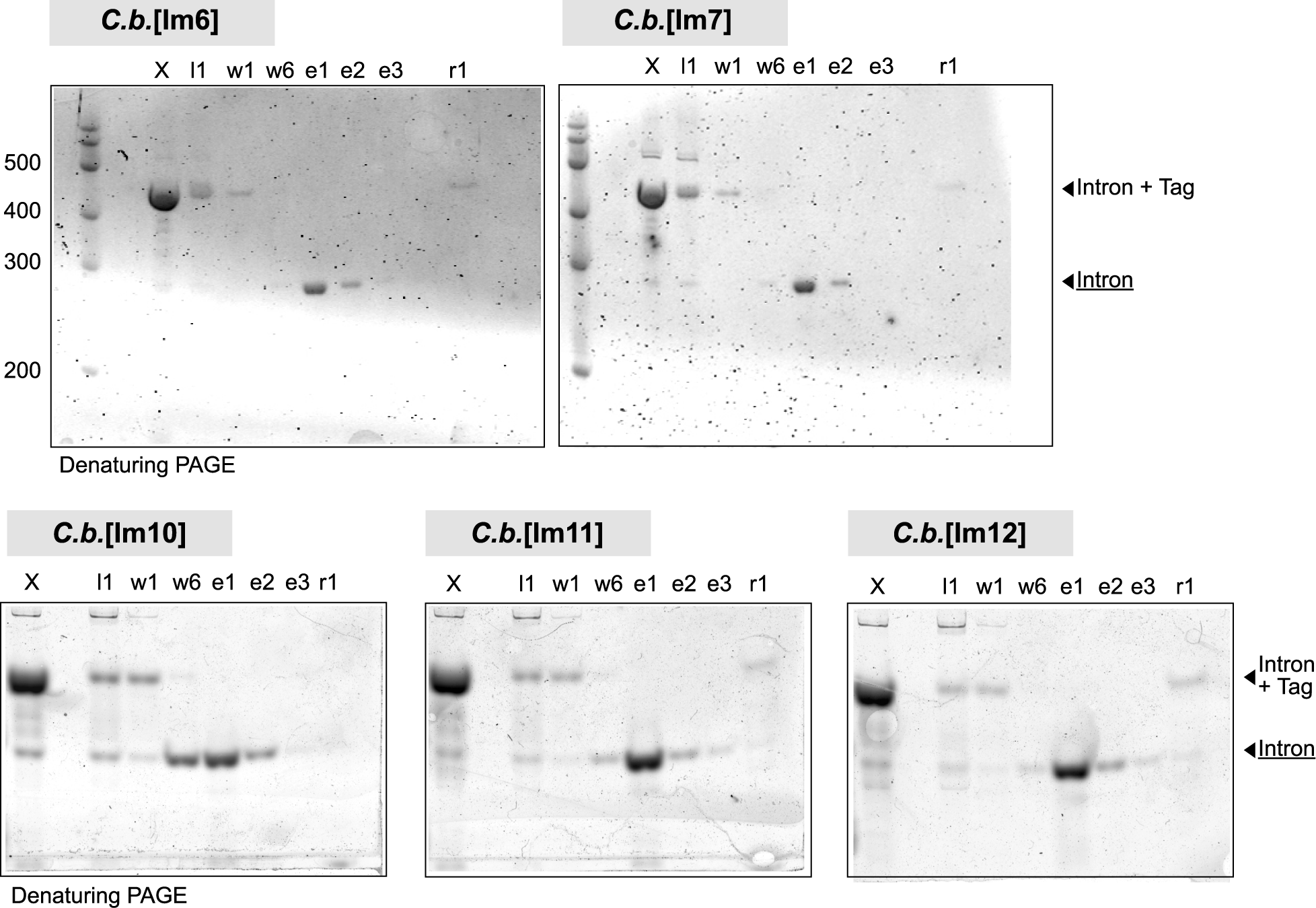

Supplement: Figure S2 — Purification assays of the C.b.[Im6], C.b.[Im7], C.b.[Im10], C.b.[Im11] and C.b.[Im12] variants. These variants were purified using the large-scale purification method (purification of 10-mL transcription reaction mixes). The assays were visualized on 6% denaturing PAGE, and stained using SYBR Green II. Labels above each lane refer to purification steps detailed in Figures 1B and 3. (0.97 MB TIF) [file pone.0006740.s003.tif]

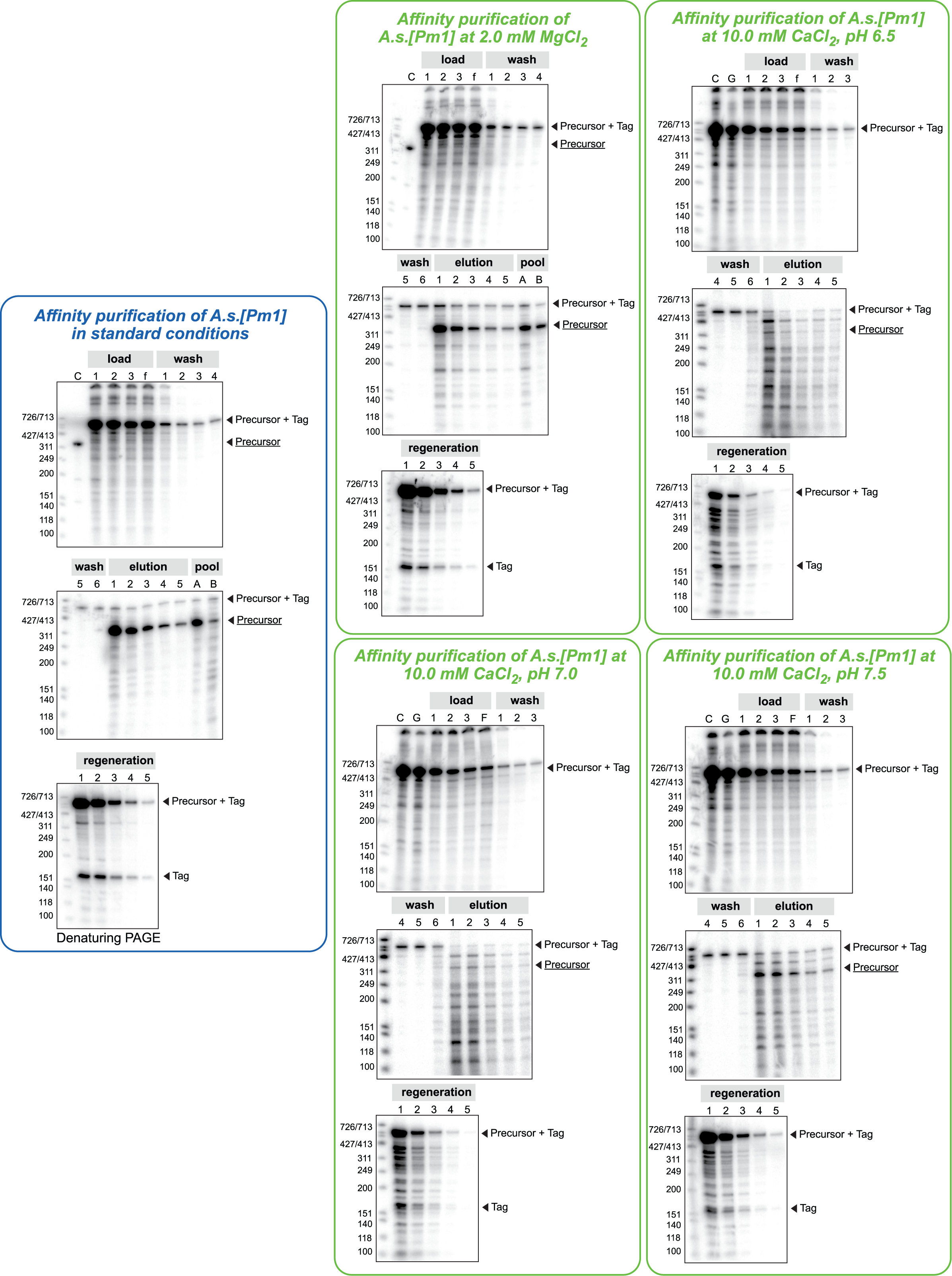

Supplement: Figure S3 — Purification assays of the A.s.[Pm1] variant at lower MgCl2 concentration, using CaCl2 in place of MgCl2, and at pH 6.5–8.0. The results are visualized by denaturing PAGE as in Figure 3; lanes are labeled according to the purification steps specified in Figure 1B. (4.10 MB TIF) [file pone.0006740.s004.tif]

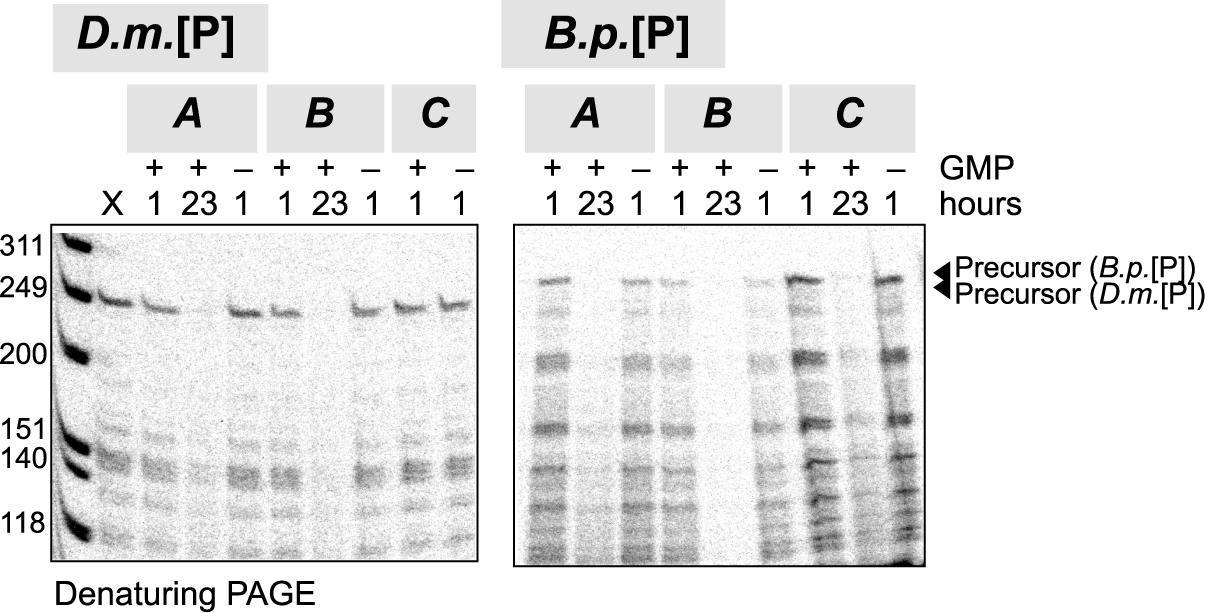

Supplement: Figure S4 — Self-splicing assays of the D.m.[P] and B.p.[P] variants. The self-splicing activity was tested under five different buffer conditions (only three shown; see Methods and [21]). (0.33 MB TIF) [file pone.0006740.s005.tif]

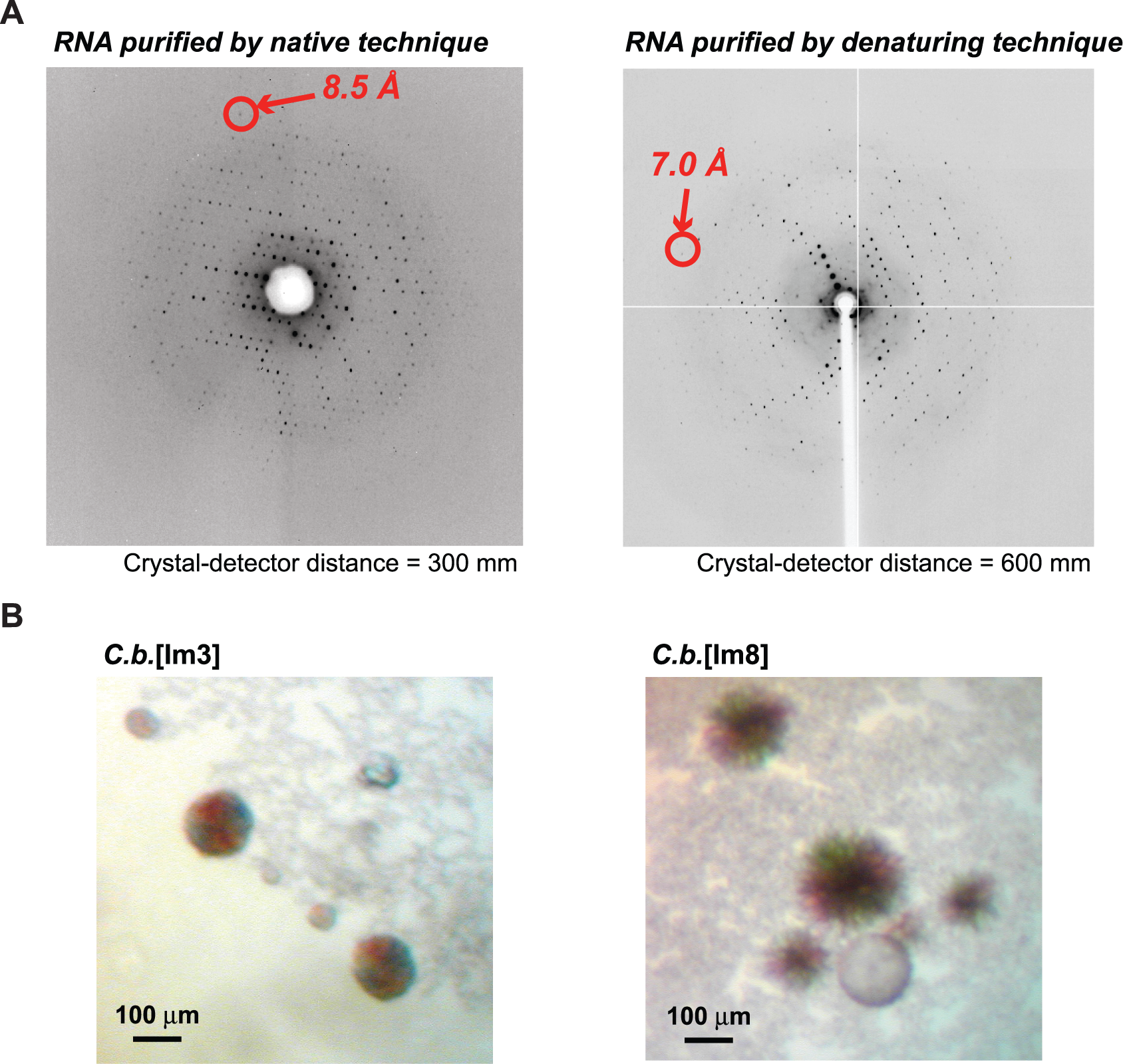

Supplement: Figure S5 — Diffraction patterns of C.b.[Im11] variants and crystalline materials obtained for the C.b.[Im3] and C.b.[Im8] variants. (A) Diffraction patterns of crystals grown using either the natively purified RNA (left), or the RNA purified by denaturing PAGE (right). The 7.0–8.5 angstrom resolution typically obtained for these crystals is indicated in red. (B) Spherulites (left) and urchins (right) obtained with the PAGE-purified C.b.[Im3] and C.b.[Im8] variants, respectively. (3.66 MB TIF) [file pone.0006740.s006.tif]

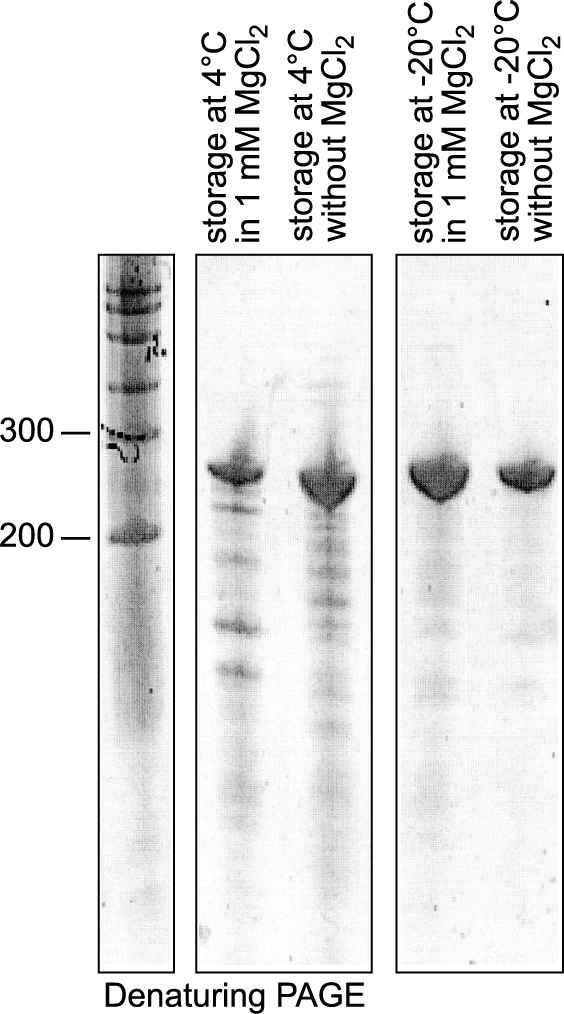

Supplement: Figure S6 — Analysis of the C.b.[Im11] RNA stock after eight weeks of storage in various conditions. The RNA was in 10 mM Na cacodylate pH 5.0 in all cases. Approximately 10 pmoles of RNA were loaded in each lane. The assays were visualized on 6% denaturing PAGE, and stained using SYBR Green II. (0.29 MB TIF) [file pone.0006740.s007.tif]

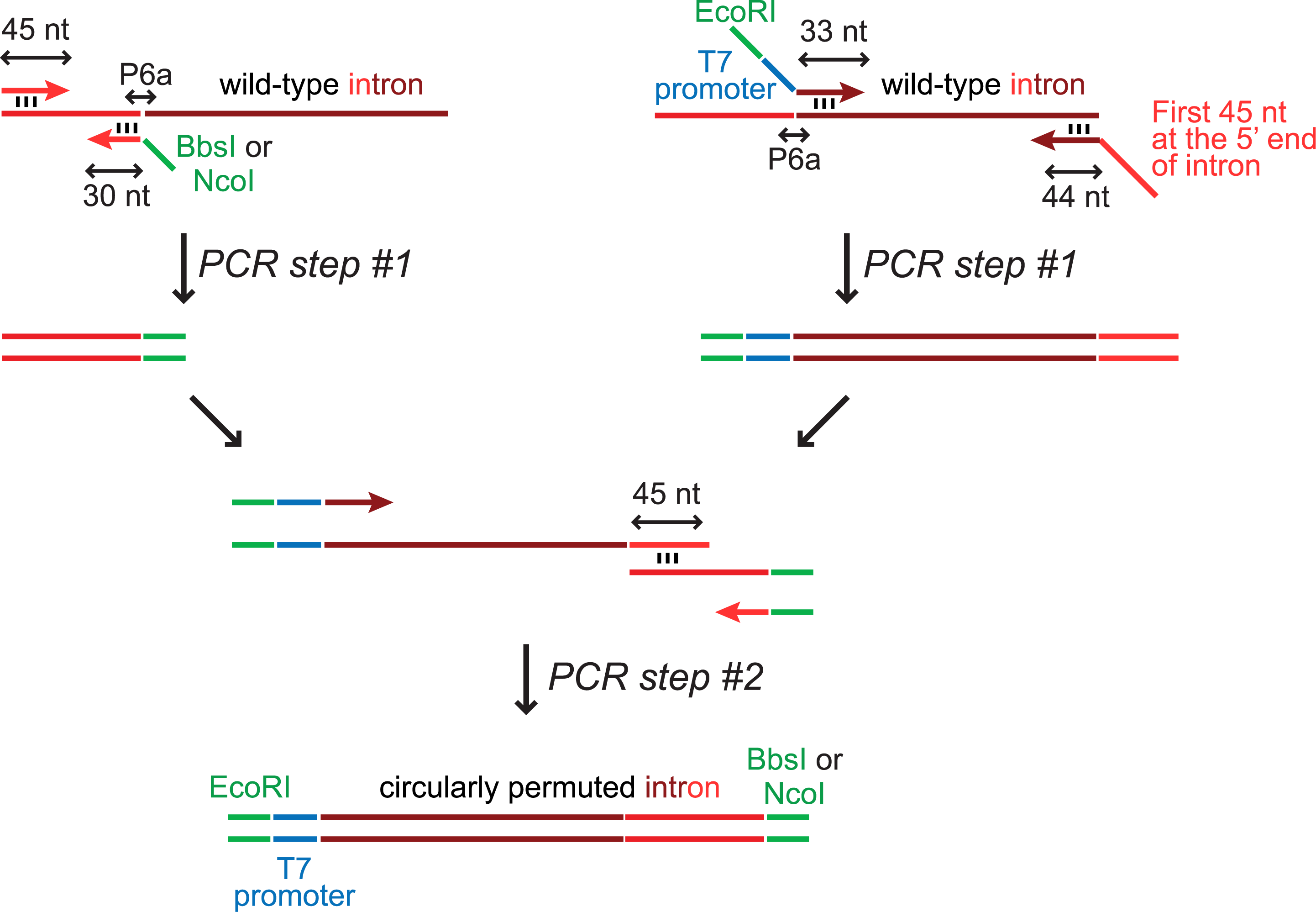

Supplement: Figure S7 — A two-step PCR strategy to circularly permute the 5′ and 3′ ends of the C.b. intron into the P6a region. During the first PCR step, the wild-type C.b. intron (colored from the wild-type 5′ end to the P6a helix (red), and from the P6a helix to the wild-type 3′ end (brown)) is used as a template for two independent PCR rounds that contain different sets of primers. During the second PCR step, the product of each of these reactions is combined to suitable primers from step #1 in order to amplify the expected circularly permuted C.b. intron. The incorporated T7 promoter and restriction sites suitable for cloning are colored as in Figure 1. (0.53 MB TIF) [file pone.0006740.s008.tif]

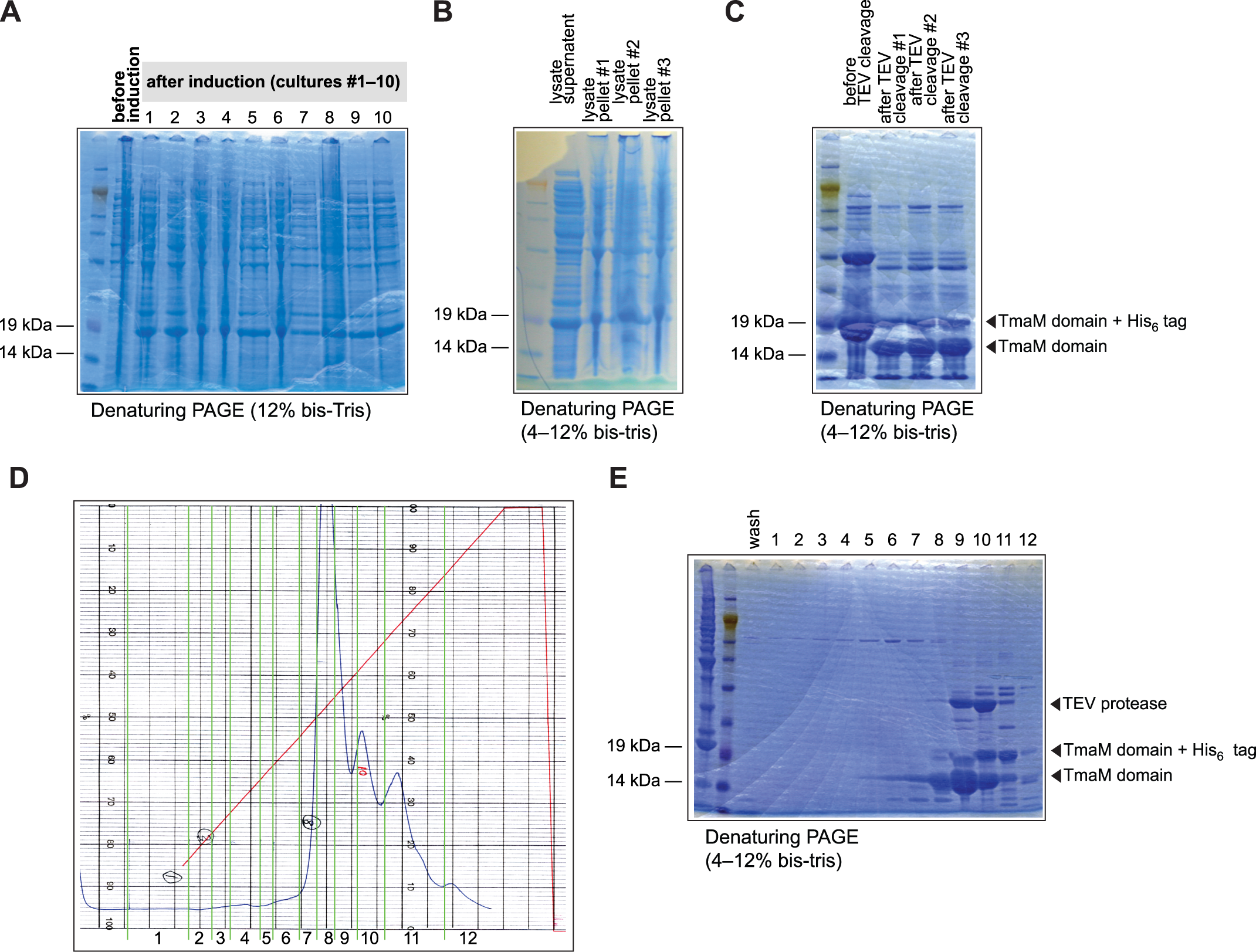

Supplement: Figure S8 — Expression and purification of the TmaM-domain protein. (A) Cells prior and post-induction with 1.0 mM IPTG (ten 750-mL cultures were grown for 12 h at 31°C in LB medium). (B) Supernatent fraction and pellets of three cell lysates. (C) Fraction of protein eluted from Ni2+-affinity column and after TEV cleavage (three fractions were cleaved for 16 h at 25°C by a 1∶100 ratio (by mass) of TEV protease, in 10 mM DTT). (D) Chromatogram after purification of the TmaM-domain on the SP-Sepharose column (12 fractions were collected, as indicated in green). The protein eluted around 0.55 M NaCl in 10 mM MES pH 6.0. (E) Corresponding elution fractions. Fractions #8 and 9 were pooled, dialysed and coupled to the activated support (Affigel-10; Bio-Rad #153-6046). Gels shown in panels A, B, C, and E were from SDS-PAGE using 4–12% or 12% acrylamide (as specified under each gel), and were stained using SimplyBlue SafeStain (Invitrogen #LC6060); ladders: SeeBlue Plus 2 ((A)–(E); Invitrogen #LC5925), BenchMark Pre-stained ((E) only; Invitrogen #10748-010). (3.29 MB TIF) [file pone.0006740.s009.tif]
